# Effects of Intra-BLA Administration of PPAR Antagonists on Formalin-Evoked Nociceptive Behaviour, Fear-Conditioned Analgesia, and Conditioned Fear in the Presence or Absence of Nociceptive Tone in Rats

**DOI:** 10.3390/molecules27062021

**Published:** 2022-03-21

**Authors:** Jessica C. Gaspar, Bright N. Okine, David Dinneen, Michelle Roche, David P. Finn

**Affiliations:** 1 Pharmacology and Therapeutics, National University of Ireland Galway, H91 W5P7 Galway, Ireland; jeccgaspar@gmail.com (J.C.G.); bnokine@gmail.com (B.N.O.); ddinneen01@qub.ac.uk (D.D.); 2 Galway Neuroscience Centre, National University of Ireland Galway, H91 W5P7 Galway, Ireland; michelle.roche@nuigalway.ie; 3 Centre for Pain Research, National University of Ireland Galway, H91 W5P7 Galway, Ireland; 4 Physiology, National University of Ireland Galway, H91 W5P7 Galway, Ireland

**Keywords:** PPAR, fear conditioning, fear-conditioned analgesia, nociception, basolateral amygdala, GW6471, GSK0660, GW9662

## Abstract

There is evidence for the involvement of peroxisome proliferator-activated receptors (PPARs) in pain, cognition, and anxiety. However, their role in pain–fear interactions is unknown. The amygdala plays a key role in pain, conditioned fear, and fear-conditioned analgesia (FCA). We investigated the effects of intra-basolateral amygdala (BLA) administration of PPARα, PPARβ/δ, and PPARγ antagonists on nociceptive behaviour, FCA, and conditioned fear in the presence or absence of nociceptive tone. Male Sprague-Dawley (SD) rats received footshock (FC) or no footshock (NFC) in a conditioning arena. Twenty-three and a half hours later, rats received an intraplantar injection of formalin or saline and, 15 min later, intra-BLA microinjections of vehicle, PPARα (GW6471) PPARβ/δ (GSK0660), or PPARγ (GW9662) antagonists before arena re-exposure. Pain and fear-related behaviour were assessed, and neurotransmitters/endocannabinoids measured post-mortem. Intra-BLA administration of PPARα or PPARγ antagonists potentiated freezing in the presence of nociceptive tone. Blockade of all PPAR subtypes in the BLA increased freezing and BLA dopamine levels in NFC rats in the absence of nociceptive tone. Administration of intra-BLA PPARα and PPARγ antagonists increased levels of dopamine in the BLA compared with the vehicle-treated counterparts. In conclusion, PPARα and PPARγ in the BLA play a role in the expression or extinction of conditioned fear in the presence or absence of nociceptive tone.

## 1. Introduction

Peroxisome-proliferator activated receptors (PPARs) are transcription factors and part of the nuclear hormone superfamily of receptors. There are three described isoforms: PPARα, PPARβ/δ, and PPARγ [1]. Endogenous ligands at PPARs include fatty acids [2], serotonin derivatives [3], the endocannabinoid anandamide (AEA; [4,5]), and the related N-acylethanolamines (NAEs) N-palmitoylethanolamide (PEA; [6]) and N-oleoylethanolamide (OEA; [7]). PPARs are involved in many physiological processes and are targets for current in-use medicines for diabetes [8] and cholesterol lowering [9].

The amygdala is part of the limbic system and plays a key role in emotional responses including anxiety and fear [10]. The basolateral amygdala (BLA) is one of three groups of nuclei in the amygdala. It is differentiated from the two other groups—central amygdala (CeA) and cortical nuclei—on account of its connections, embryonic origin, and cytoarchitecture [11]. The BLA receives input from several brain regions including the hippocampus, hypothalamus, cerebral cortex, ventral tegmental area (VTA), and thalamus [11,12,13]. It also has an extensive efferent network, which includes the CeA, periaqueductal grey (PAG), ventral striatum, dorsal striatum (caudate-putamen), hippocampus, and others [11,12,13]. The BLA has a central role in emotional behaviour, particularly, fear conditioning. It has been shown that lesions [14,15,16,17,18,19,20] or inactivation by muscimol [21,22,23,24,25] of the BLA impaired acquisition and the expression of fear conditioning. Inactivation of the BLA also affects fear extinction [26]. Some other general behaviours such as rearing and distance moved in an open field arena have also been shown to be affected by electrolytic lesions of the BLA [27]. Moreover, reversible blockade of the BLA by lidocaine infusions results in increased rearing responses and locomotor activity after amphetamine administration [28]. The GABAergic [29], glutamatergic [30,31], serotoninergic [32], dopaminergic [33], and endocannabinoid [34] systems have all been shown to participate in this modulatory role of the BLA.

Pain is a complex condition with sensory-motor, emotional, and cognitive aspects. The amygdala is part of both the descending pain pathway and the limbic system and is involved in the emotional-affective aspect of pain. Neurons in the BLA respond to chronic [35] and acute [36,37] noxious stimuli and the pharmacological deactivation of the BLA reduced pain-related behaviour [35]. Additionally, intraplantar injection of formalin increased *c-fos* expression in the BLA [38].

Pain and fear modulate one another in a reciprocal manner. The phenomenon known as fear-conditioned analgesia (FCA), in which a fearful stimulus causes a significant suppression in pain response, is an example of the influence of fear on pain. In turn, pain can regulate fear responses. Post-traumatic stress disorder (PTSD) symptoms tend to be more pronounced in patients with chronic pain [39]. Moreover, patients with chronic pain are twice as likely to develop phobias [40]. PPAR isoforms are expressed in brain regions that play an important role in pain and fear/anxiety such as the amygdala [41], PFC [41,42,43], hippocampus [42,44], and PAG [45].

Studies have indicated a likely role for PPARs in pain (see [46] for review), but the role of PPARs expressed in the amygdala in pain has not been examined yet. There is some evidence that PPARγ blockade or knockout has anxiogenic effects in mice [44,47]. However, whether PPARβ/δ and PPARα modulate anxiety or fear remains unexplored. Moreover, the role of PPARs expressed in the BLA in interactions between pain and fear has not been investigated yet. Previous work from our research group has provided evidence that PPAR blockade can potentiate conditioned fear-related behaviour in the presence of nociceptive tone [48], but the brain regions mediating these effects remain to be elucidated.

Thus, a better understanding of the mechanisms underlying anxiety, fear, pain, and their mutual modulation, particularly the role of the PPAR signalling system in these phenomena, is necessary. In this study, we investigated the hypothesis that PPARs expressed in the BLA modulate tonic inflammatory pain, fear, and their interaction. Specifically, we examined the effects of intra-BLA administration of GW6471 (PPARα antagonist), GSK0660 (PPARβ/δ antagonist), and GW9662 (PPARγ antagonist) on formalin-induced nociceptive behaviour and FCA in rats. We also investigated the effects of intra-BLA administration of these antagonists on conditioned-fear related behaviour, both in the presence and absence of nociceptive tone in rats. In addition, associated alterations in levels of neurotransmitters, endocannabinoids, and NAEs in the BLA were analysed. Furthermore, differences in the levels of neurotransmitters, endocannabinoids, and NAEs in FC and NFC rats that received either formalin or saline injection were also analysed.

## 2. Results

### 2.1. Experiment 1: Effects of Intra-BLA Administration of PPAR Antagonists on Formalin-Evoked Nociceptive Behaviour, Fear-Conditioned Analgesia, and Conditioned Fear in the Presence of Nociceptive Tone in Rats

#### 2.1.1. Intra-BLA Administration of GW6471, GSK0660, and GW9662 Had No Effect on Formalin-Evoked Nociceptive Behaviour or FCA

After histological verification, 75% of the rats had both injections correctly placed within the borders of both BLA. Additionally, 4% had one of the injections in the BLA and the other outside BLA borders (see Supplementary Figure S1 for details). The remaining 21% were placed in the CeA, basomedial amygdala (BMA), or ventral endopiriform nucleus. The data analysed were derived only from rats where intracerebral microinjections were accurately placed in the BLA.

**Figure 1:**
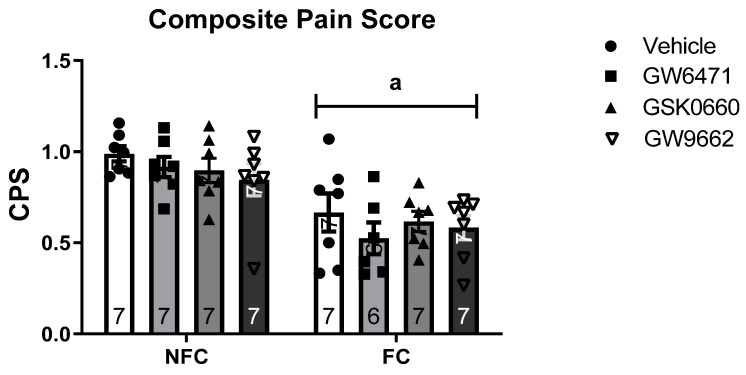
Effects of intra-BLA administration of selective PPARα, PPARβ/δ, and PPARγ antagonists on formalin-evoked nociceptive behaviour (CPS; composite pain score) in non-fear conditioned (NFC) and fear conditioned (FC) rats. Data are expressed as mean ± S.E.M (*n* = 6–7 rats per group). According to a 2-way ANOVA (^a^ *p* < 0.001), significant overall effect of fear conditioning.

**Figure 2:**
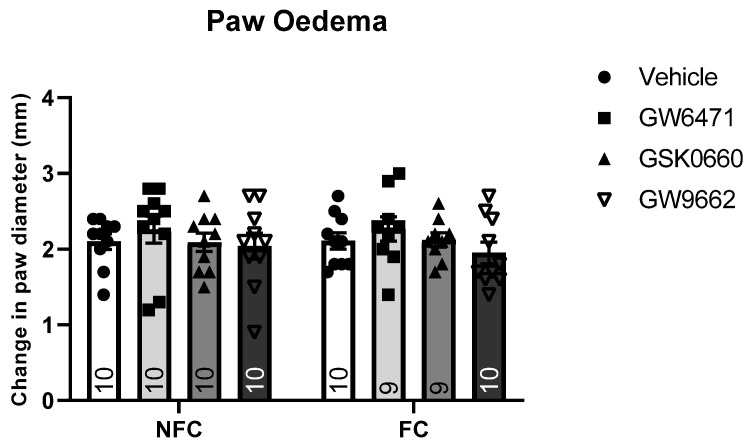
Effects of intra-BLA administration of selective PPARα, PPARβ/δ, and PPARγ antagonists on paw oedema. Paw oedema was assessed by measuring the change in the diameter of the right hind paw immediately before, and 60 min after, formalin administration. Data are expressed as mean ± S.E.M, *n* = 9–10 rats per group.

Intraplantar administration of formalin into the right hind paw produced robust nociceptive behaviour, as evidenced by the CPS (Figure 1). Two-way ANOVA revealed a significant main effect of fear conditioning [F (1, 54) = 35.264, ^a^ *p* < 0.05], but not of treatment [F (3, 47) = 0.987, *p* > 0.05] or treatment × conditioning [F (2, 54) = 0.304, *p* > 0.05], on nociceptive behaviour (Figure 2). However, post hoc pairwise analysis with Student Newman–Keuls test revealed there were no significant differences between groups. There were no significant effects of fear-conditioning [F (1, 70) = 0.011, *p* > 0.05], treatment [F (3, 70) = 0.296, *p* > 0.05], or treatment × conditioning [F (2, 70) = 0.078, *p* > 0.05] on formalin-induced paw oedema (Figure 2).

#### 2.1.2. Intra-BLA Administration of GW6471, GSK0660, and GW9662 Increases Fear-Related Behaviour in Formalin-Treated Rats

**Figure 3:**
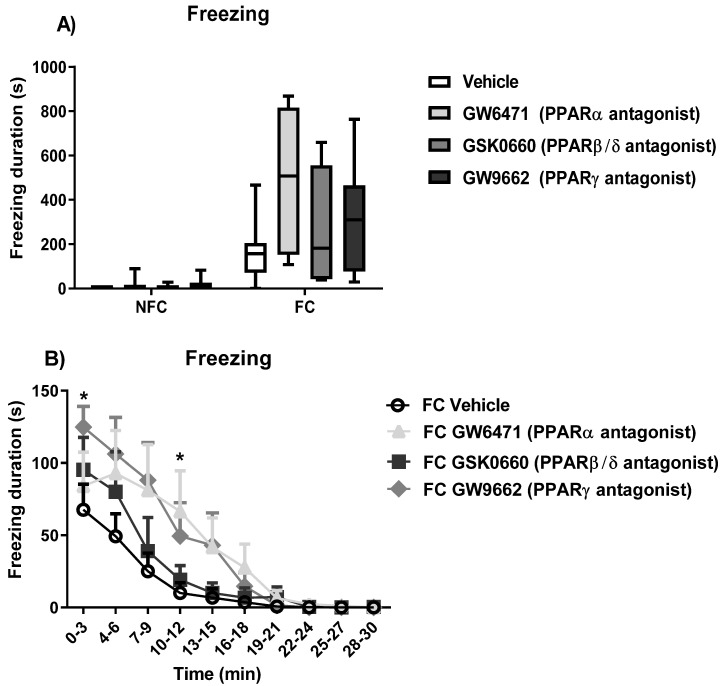
Effects of intra-BLA administration of selective PPARα, PPARβ/δ, and PPARγ antagonists on freezing duration over the total trial period (**A**) and as 3-min time bins (**B**) in non-fear conditioned (NFC) and fear conditioned (FC) rats. Post hoc analysis indicated a significant increase at 0–3 min for FC GW9662-treated rats (* *p* < 0.05, vs. FC vehicle), and FC GW6471-treated rats at 10–12 min (* *p* < 0.05, vs. FC vehicle). Data are expressed as the median with interquartile range and min/max (**A**) and mean ± S.E.M. (**B**) (*n* = 7–9 rats per group).

Kruskal–Wallis test revealed a significant difference between the groups on total duration of freezing [χ^2^ (7) = 34.508, *p* < 0.001] (Figure 3A). However, there were no significant differences with Dunn’s post hoc pairwise analysis.

In an analysis of the data as 3-min time bins, two-way repeated measures ANOVA revealed a significant effect of fear conditioning [F (1, 47) = 37.456, *p* < 0.001], time [F (2.251, 105.816) = 38.350, * *p* < 0.001), and fear conditioning × time [F (2.251, 105.816) = 35.556, *p* < 0.001] on freezing duration (Figure 3B; NFC groups not shown for clarity of presentation, but are presented in Supplementary Figure S2). Post hoc analysis by Student Newman–Keuls test indicated a significant increase in the duration of freezing in FC GW6471 vs. FC Vehicle at 10–12 min (^$^ *p* < 0.05) and at 0–3 min for FC GW9662 vs. FC Vehicle (^#^ *p* < 0.05) (Figure 3B). There were no significant effects of drug treatment on freezing across time in NFC rats (Supplementary Figure S2). There were no significant effects of treatment [F (3, 47) = 1.750, *p* > 0.05], treatment × conditioning [F (3, 47) = 1.591, *p* > 0.05], time × treatment [F (6.754, 105.816) = 1.538, *p* > 0.05], time × conditioning [F (2.251, 105.816) = 35.556, *p* > 0.05], and time × conditioning × treatment [F (6.754, 105.8160 = 1.372, *p* > 0.05] on freezing duration.

**Figure 4:**
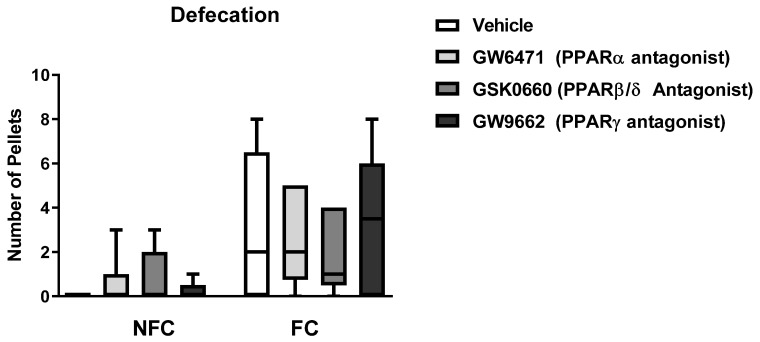
Effects of fear conditioning and intra-BLA administration of selective PPARα, PPARβ/δ, and PPARγ antagonists on defecation. Data are expressed as median with interquartile range and min/max (*n*= 7–9 rats per group).

Kruskal–Wallis test also revealed a significant difference in defecation among all groups [χ^2^ (7) = 24.023, *p* < 0.01] (Figure 4). However, post hoc pairwise analysis with Dunn’s test did not reveal significant differences between pairs of groups.

#### 2.1.3. Intra-BLA Administration of GW6471, GSK0660, and GW9662 Does Not Affect General/Motor Behaviour

**Figure 5:**
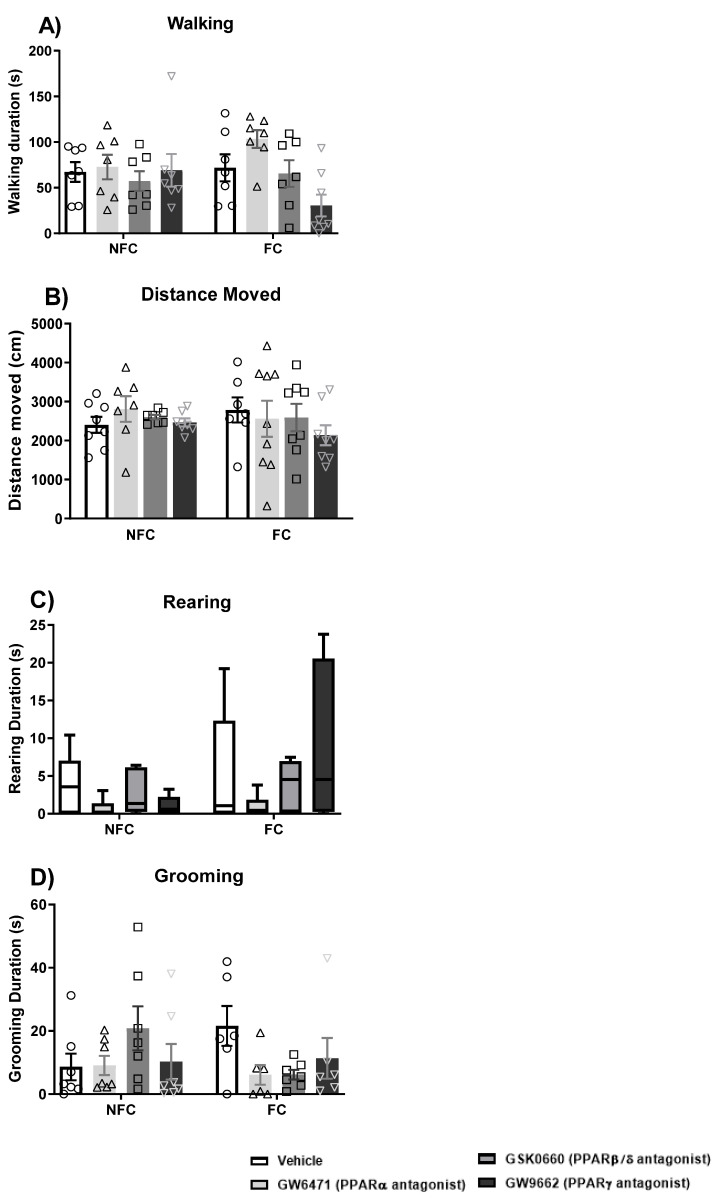
Effects of fear-conditioning and intra-BLA administration of selective PPARα, PPARβ/δ, and PPARγ antagonists on walking duration (**A**), distance moved (**B**), grooming duration (**C**), and rearing duration (**D**). Data are expressed as mean ± S.E.M. (**A**,**B**,**D**) or median with interquartile range and min/max (**C**), each symbol represents one individual, *n* = 7–9 rats per group.

The effects of fear-conditioning and intra-BLA administration of PPAR antagonists on motor behaviour were also assessed (Figure 5). Neither fear conditioning nor PPAR antagonists had any significant effect on walking (two-way ANOVA [F (3, 53) = 0.294, *p* > 0.05], fear conditioning [F (1, 53) = 0.251, *p* > 0.05], treatment × conditioning [F (3, 53) = 1.42]; Figure 5A), rearing (Kruskal–Wallis [χ^2^ (7) = 5.685, *p* > 0.05]; Figure 5C), grooming (two-way ANOVA [F (3, 53) = 0.043, *p* > 0.05], fear conditioning [F (1, 53) = 0.380, *p* > 0.05], and treatment × conditioning [F (3, 53) = 0.268, *p* > 0.05); Figure 5D) or distance moved (two-way ANOVA [F (3, 53) = 0.591, *p* > 0.05], fear conditioning [F (1, 53) = 0.056, *p* > 0.05], and treatment × conditioning [F (3, 53) = 0.532, *p* > 0.05]; Figure 5B) in either the NFC or FC rats.

#### 2.1.4. Effect of Fear Conditioning and PPAR Antagonist Administration on Neurotransmitter Levels in the Basolateral Amygdala (BLA)

**Figure 6:**
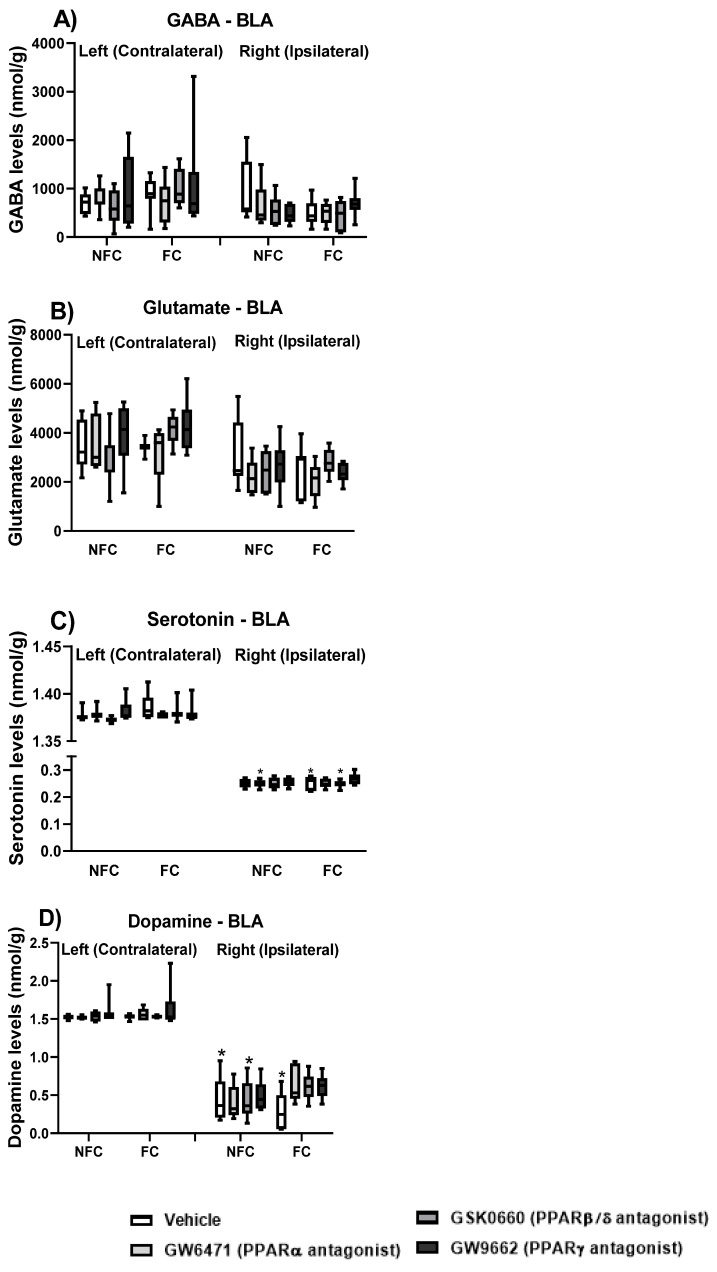
Effects of fear-conditioning and intra-BLA administration of PPARα, PPARβ/δ, and PPARγ antagonists on the levels of GABA (**A**), glutamate (**B**), serotonin (**C**), and dopamine (**D**). Post hoc analysis indicated that dopamine levels were significantly lower in the right BLA of NFC Vehicle, FC Vehicle, and NFC GSK0660 rats compared to their left counterparts (* *p* < 0.05). Post hoc analysis also indicated that levels of serotonin were lower in the right BLA of NFC GW6471, FC Vehicle, and FC GSK0660 rats compared to their left side counterparts (* *p* < 0.05). Data are expressed as the median with interquartile range and min/max (*n* = 7–9 rats per group).

In order to further explore the neurochemical mechanisms underlying the effect of PPAR antagonism on fear memory, the levels of neurotransmitters (glutamate, GABA, dopamine and serotonin) in the right (ipsilateral) and left (contralateral, in relation to the formalin injection, which was into the right hind paw) BLA were measured in all groups (Figure 6).

Kruskal–Wallis comparisons revealed a significant difference between groups (χ^2^ (15) = 84.814, *p* < 0.001) in serotonin levels in the BLA (Figure 6C). Post hoc analysis with Dunn’s test indicated that the levels of serotonin were significantly lower in the right BLA of NFC GW6471, FC Vehicle, and FC GSK0660 rats compared to their left side counterparts (* *p* < 0.05). When each side was analysed separately, Kruskal–Wallis showed a significant difference among the groups in the left [χ^2^ (7) = 16.134, *p* < 0.05], but not in the right [χ^2^ (7) = 4.713, *p* > 0.05] side in serotonin levels in the BLA. However, post hoc pairwise comparisons with Dunn’s test did not reach statistical significance. The Kruskal–Wallis test revealed a significant difference between groups (χ^2^ (15) = 90.526, *p* < 0.001) in dopamine levels in the BLA (Figure 6D). Post hoc analysis with Dunn’s test indicated that the levels of dopamine were significantly lower in the right BLA of NFC Vehicle, FC Vehicle, and NFC GSK0660 rats compared to their left counterparts (* *p* < 0.05). When each side was analysed separately, Kruskal–Wallis did not show any significant difference between groups in the right [χ^2^ (7) = 11.912, *p* > 0.05] or in the left [χ^2^ (7) = 1.796, *p* > 0.05] sides in dopamine levels in the BLA.

The Kruskal–Wallis test did not show any significant difference between groups (χ^2^ (15) = 20.669, *p* > 0.05) in GABA levels in the BLA (Figure 6A). When each side was analysed separately, Kruskal–Wallis did not show any significant difference between groups in the right [χ^2^ (7) = 6.288, *p* > 0.05] or in the left [χ^2^ (7) = 5.291, *p* > 0.05] sides in the GABA levels in the BLA. Kruskal–Wallis comparisons revealed a significant difference between groups (χ^2^ (15) = 39.443, *p* < 0.01) in glutamate levels in the BLA (Figure 6B). However, post hoc analysis with Dunn’s test did not reach statistical significance. When each side was analysed separately, Kruskal–Wallis did not show any significant difference between group in the right [χ^2^ (7) = 5.432, *p* > 0.05] or in the left [χ^2^ (7) = 9.575, *p* > 0.05] sides in glutamate levels in the BLA.

#### 2.1.5. Effect of Fear Conditioning and PPAR Antagonist Administration on Endocannabinoids and NAE Levels in the Basolateral Amygdala (BLA)

We also investigated endogenous ligands for PPARs (AEA, PEA, and OEA) in the BLA, and checked for possible effects of PPAR antagonism and intraplantar formalin injection on their levels.

**Figure 7:**
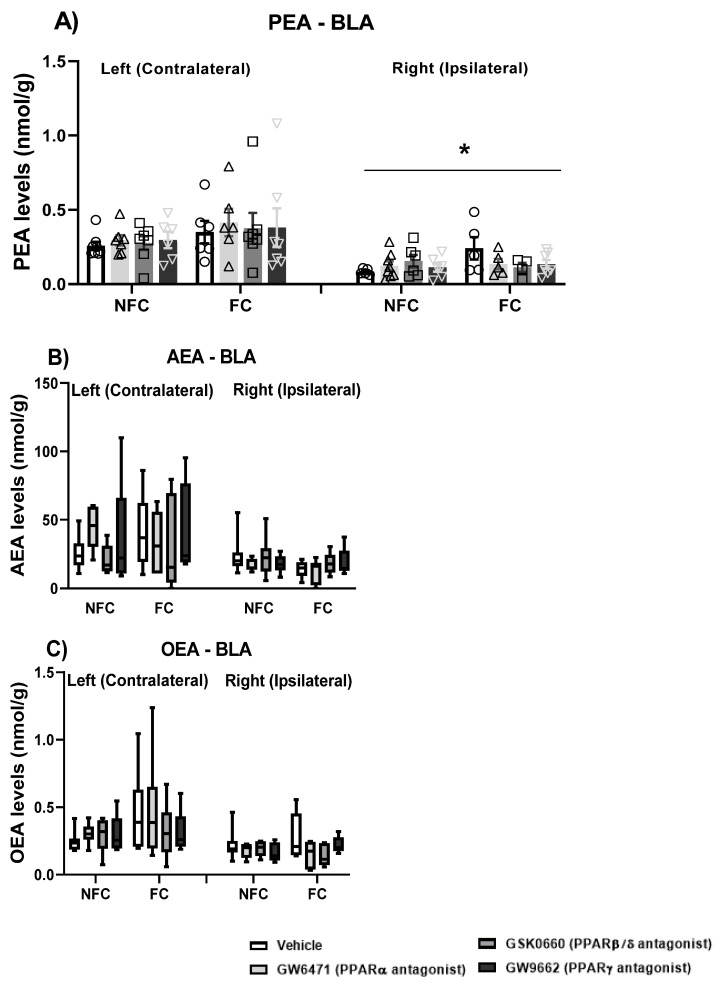
Effects of fear-conditioning and intra-BLA administration of selective PPARα, PPARβ/δ, and PPARγ antagonists on the levels of PEA (**A**), AEA (**B**), and OEA (**C**). Two-way ANOVA revealed a significant effect of side on PEA levels (* *p* < 0.05). Data are expressed as mean ± S.E.M (**A**) or median with interquartile range and min/max (**B**,**C**), each symbol represents one individual, (*n* = 7–9 rats per group).

Three-way ANOVA revealed an effect of side [F (1, 84) = 49.888, * *p* < 0.001] and fear conditioning [F (1, 84) = 4.298, *p* < 0.05] on PEA levels in the BLA (Figure 7A). Post hoc pairwise analysis with Student Newman–Keuls test did not show any significant statistical differences. There were no significant effects of treatment [F (3, 84) = 0.068, *p* > 0.05], treatment × conditioning [F (3, 84) = 0.669, *p* > 0.05], treatment × side [F (3, 84) = 0.344, *p* > 0.05], conditioning × side [F (1, 84) = 0.074, *p* > 0.05], and treatment × conditioning × side [F (3, 84) = 0.656, *p* > 0.05] on PEA levels. When each side was analysed separately, two-way ANOVA did not show any significant effect of treatment, conditioning, or their interaction on either the left or right BLA.

Kruskal–Wallis comparisons did not show any significant difference between groups (χ^2^ (15) = 22.173, *p* > 0.05) in AEA levels in the BLA (Figure 7B). When each side was analysed separately, Kruskal–Wallis did not show any significant difference between groups in the right [χ^2^ (7) = 4.721, *p* > 0.05] or in the left [χ^2^ (7) = 6.548, *p* > 0.05] sides in the AEA levels in the BLA.

Kruskal–Wallis comparisons revealed a significant difference between groups (χ^2^ (15) = 31.454, *p* < 0.01) in OEA levels in the BLA (Figure 7C). However, post hoc analysis with Dunn’s test did not reach statistical significance. When each side was analysed separately, the Kruskal–Wallis test did not show any significant difference between groups in the right [χ^2^ (7) = 6.672, *p* > 0.05] or in the left [χ^2^ (7) = 4.598, *p* > 0.05] sides in the OEA levels in the BLA.

### 2.2. Experiment 2: Effects of Intra-BLA Administration of PPAR Antagonists on Conditioned Fear in the Absence of Nociceptive Tone in Rats

#### 2.2.1. *Intra-BLA Administration of PPAR Antagonists Had No Effect on* *Composite Pain Score in Saline-Injected Rats*

After histological verification, 73% of the rats had both injections correctly placed within the borders of both BLA. Additionally, 7% had one of the injections in the BLA and the other outside BLA borders (see Supplementary Figure S3 for details). The remaining 20% were placed in the CeA, basomedial amygdala (BMA), or ventral endopiriform nucleus. The data analysed were derived only from rats where intracerebral microinjections were accurately placed in the BLA.

**Figure 8:**
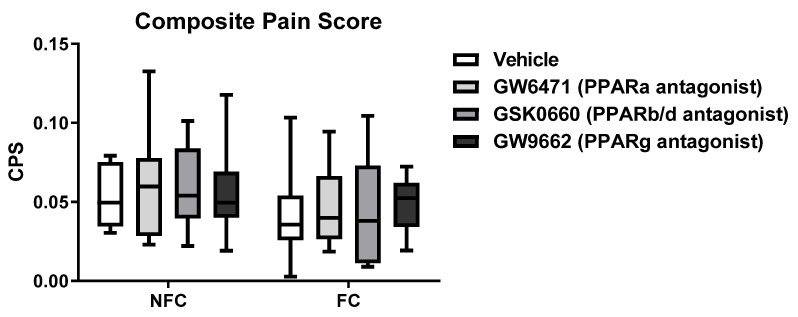
Effects of intra-BLA administration of selective PPARα, PPARβ/δ, and PPARγ antagonists on the composite pain score in non-fear conditioned (NFC) and fear conditioned (FC) rats that received an intraplantar injection of saline. Composite pain score was calculated as (pain 1 + 2 × [pain 2])/total duration of analysis period (see for further information in the Material and Methods). Kruskal–Wallis showed no significant difference between groups [χ^2^ (7) = 4.241, *p* > 0.05]. Data are expressed as median with interquartile range and min/max (*n* = 8–10 rats per group).

**Figure 9:**
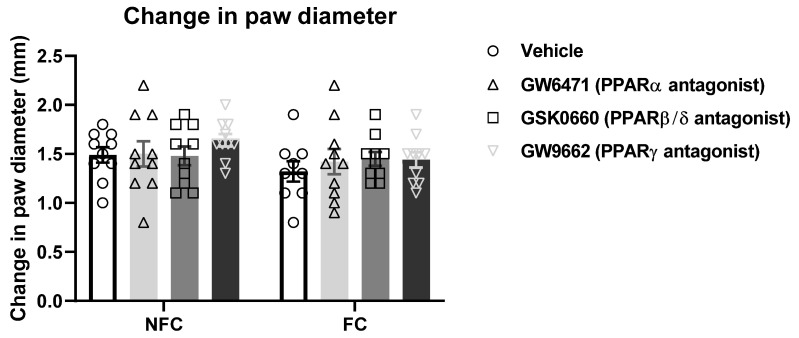
Effects of intra-BLA administration of selective PPARα, PPARβ/δ, and PPARγ antagonists on saline-evoked changes in the hind paw diameter in non-fear conditioned (NFC) and fear conditioned (FC) rats. The change was assessed by measuring the paw diameter immediately before, and 60 min after, saline administration. Data are expressed as mean ± S.E.M, each symbol represents one individual, *n* = 8–10 rats per group.

As expected, composite pain scores were substantially less in this experiment following intraplantar saline injection compared with Experiment 1, where the rats received intraplantar formalin injection (Figure 8). The Kruskal–Wallis test revealed no difference between groups [χ^2^ (7) = 4.241, *p* > 0.05] of rats that received an intraplantar injection of saline into the right hind paw (Figure 9). Two-way ANOVA showed that there were no significant effects of fear-conditioning [F (1, 9) = 4.364, *p* > 0.05], treatment [F (3, 27) = 0.5191, *p* > 0.05], or treatment × conditioning [F (3, 26) = 0.4741, *p* > 0.05] on paw diameter (Figure 9).

#### 2.2.2. Intra-BLA Administration of PPARs Antagonists Increases Freezing in NFC Rats

**Figure 10:**
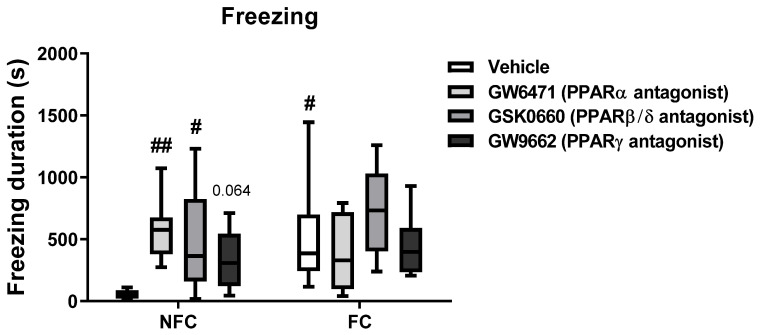
Effects of fear conditioning and intra-BLA administration of selective PPARα, PPARβ/δ, and PPARγ antagonists on total freezing duration. Post hoc indicated an increase in freezing duration in FC Vehicle rats (^#^ *p* < 0.05, vs. NFC Vehicle). The treatment with GW6471 and GSK0660 in NFC rats also increased freezing duration (^##^ *p* < 0.01 vs. NFC Vehicle; ^#^ *p* < 0.05 vs. NFC Vehicle). Treatment with GW9662 almost reached statistical significance (*p* = 0.064, vs. NFC Vehicle). Data are expressed as median with interquartile range and min/max (*n*= 7–9 rats per group).

Kruskal–Wallis revealed a significant difference between groups on the total duration of freezing [χ^2^ (7) = 18.037, *p* = 0.012] (Figure 10; the graphs for 3-min bins are available as Supplementary Figure S4). Post hoc pairwise analysis with Dunn’s test indicates a significant enhancement in freezing duration in FC Vehicle rats compared to their NFC counterparts (NFC Vehicle vs. FC Vehicle, * *p* < 0.05). The treatment with GW6471 and GSK0660 in NFC rats also increased freezing duration (NFC Vehicle vs. NFC GW6471, ** *p* < 0.01; NFC Vehicle vs. NFC GSK0660, * *p* < 0.05). The treatment with GW9662 in NFC rats narrowly failed to reach statistical significance (NFC Vehicle vs. GW9662, *p* = 0.064). These drugs had no significant effects on FC rats.

**Figure 11:**
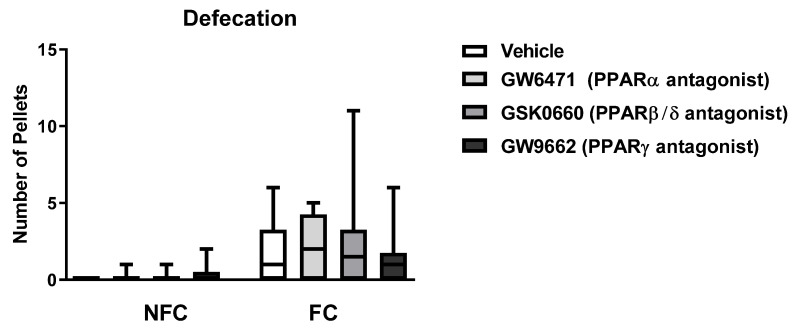
Effects of fear conditioning and intra-BLA administration of selective PPARα, PPARβ/δ, and PPARγ antagonists on defecation. Data are expressed as median with interquartile range and min/max (*n* = 7–9 rats per group).

Kruskal–Wallis analysis of the defecation data revealed a significant difference between groups [χ^2^ (7) = 23.49, *p* < 0.01] (Figure 11). Post hoc pairwise analysis with Dunn’s test did not show any significant difference between groups.

#### 2.2.3. Intra-BLA Administration of PPAR Antagonists Does Not Affect General/Motor Behaviour

**Figure 12:**
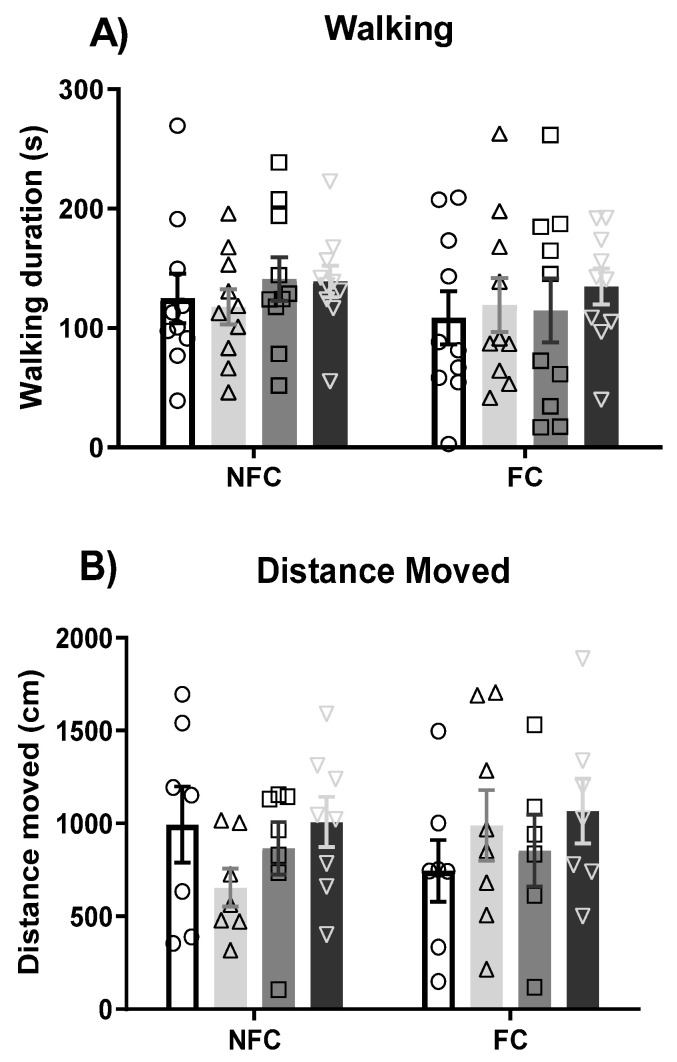

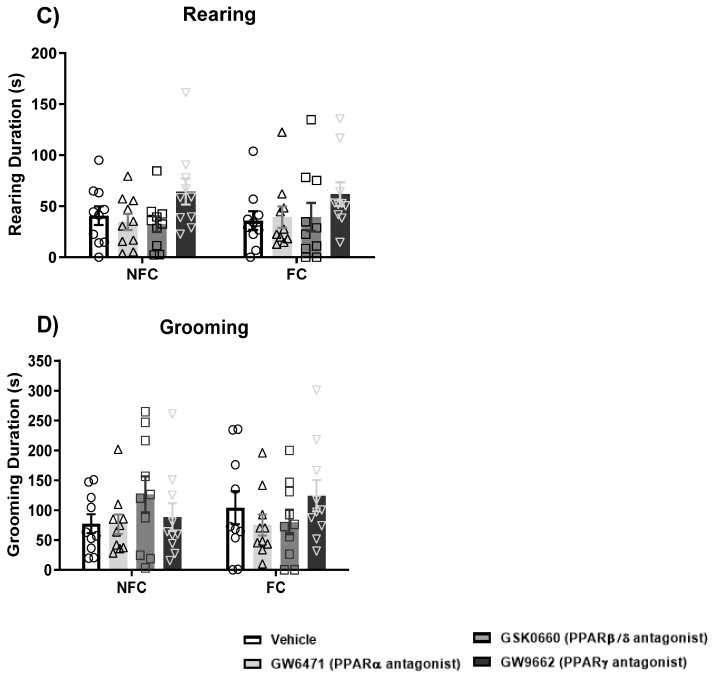
Effects of fear-conditioning and intra-BLA administration of selective PPARα, PPARβ/δ, and PPARγ antagonists on walking duration (**A**), distance moved (**B**), rearing duration (**C**), and grooming duration (**D**). Data are expressed as mean ± S.E.M.), each symbol represents one individual, (*n* = 7–9 rats per group).

The effects of fear-conditioning and intra-BLA administration PPAR antagonists on motor behaviour were also assessed (Figure 12). Neither fear conditioning nor PPAR antagonists induced any significant effect on walking (two-way ANOVA [F (3, 58) = 0.332, *p* > 0.05], fear conditioning [F (1, 58) = 0.133, *p* > 0.05], treatment × conditioning [F (3, 58) = 0.244, *p* > 0.05]; Figure 12A), distance moved (two-way ANOVA [F (3, 58) = 0.716, *p* > 0.05], fear conditioning [F (1, 58) = 0.055, *p* > 0.05], treatment × conditioning [F (3, 58) = 1.199, *p* > 0.05]; Figure 12B), grooming (two-way ANOVA [F (3, 50) = 0.628, *p* > 0.05], fear conditioning [F (1, 50) = 0.053, *p* > 0.05], and treatment × conditioning [F (3, 50) = 0.248, *p* > 0.05); Figure 12D).

Two-way ANOVA showed an effect of treatment [F (3, 50) = 3.686, *p* < 0.05] on rearing duration (Figure 12C). However, post hoc analysis with Student Newman–Keuls did not reveal significant statistical differences between groups. There were no significant effects of fear conditioning [F (1, 50) = 0.261, *p* > 0.05] and treatment × conditioning [F (3, 50) = 0.256, *p* > 0.05] on rearing duration.

#### 2.2.4. Effect of Fear Conditioning and PPAR Antagonist Administration on Neurotransmitter Levels in the Basolateral Amygdala (BLA)

**Figure 13:**
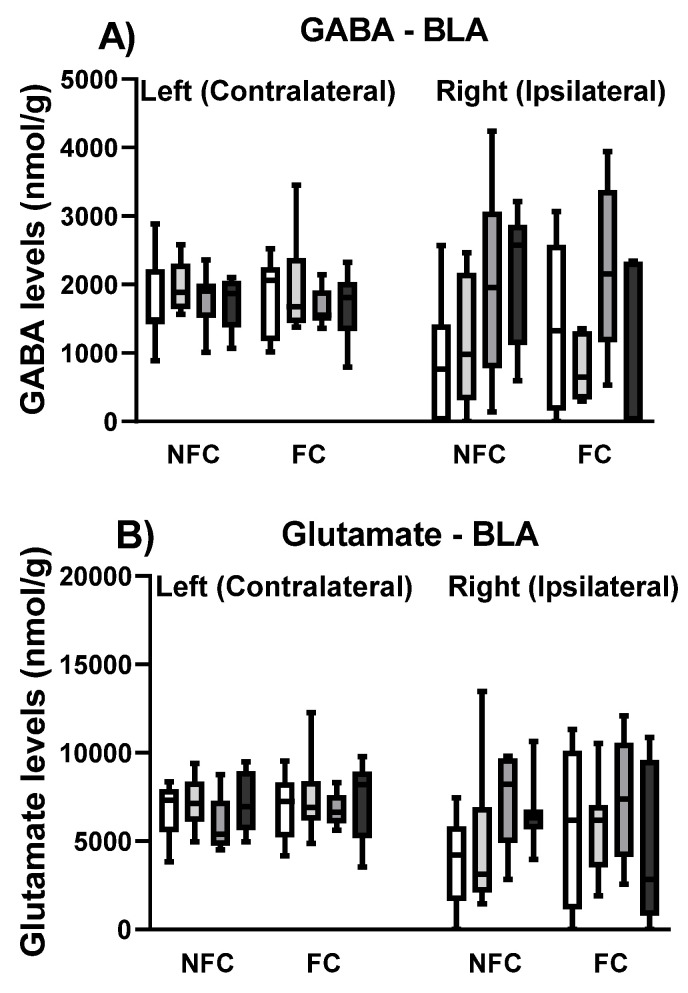

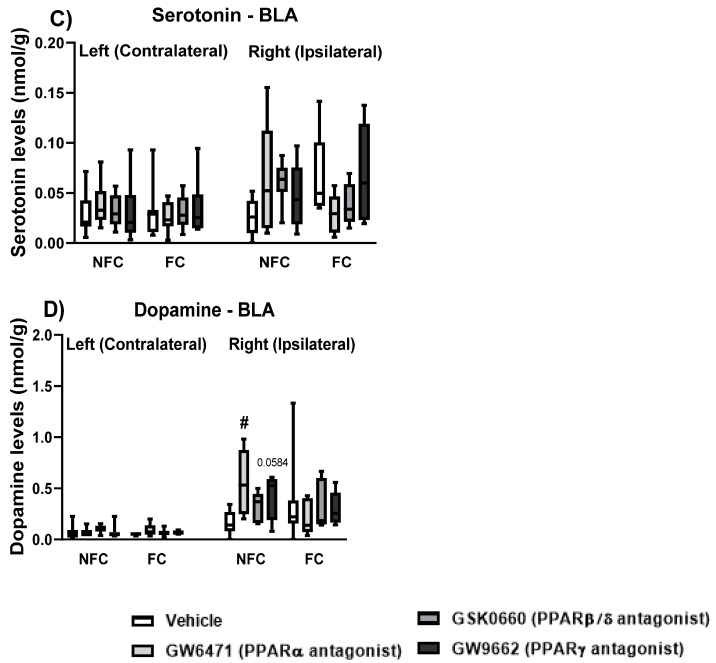
Effects of fear-conditioning and intra-BLA administration of selective PPARα, PPARβ/δ and PPARγ antagonists on the tissue levels of GABA (**A**), glutamate (**B**), serotonin (**C**), and dopamine (**D**) in the BLA. Post hoc analysis indicated that NFC rats treated with GW6471 had increased dopamine levels compared to the NFC Vehicle-treated ones (^#^ *p* < 0.05). The test also indicated a strong trend for increased levels of dopamine in the NFC GW9662-treated rats compared to the NFC vehicle-treated (*p* = 0.0584). Data are expressed as median with interquartile range and min/max (*n* = 7–9 rats per group).

As in experiment 1, we further explored the neurochemical mechanisms underlying the effect of PPAR antagonism on fear memory, now in the absence of nociceptive tone, by checking the levels of neurotransmitters (glutamate, GABA, dopamine, and serotonin) in the right (ipsilateral) and left (contralateral, in relation to the saline injection, which was into the right hind paw) BLA of all groups (Figure 13).

The Kruskal–Wallis test revealed a significant difference between groups (χ^2^ (15) = 58.963, *p* < 0.001) in dopamine levels (Figure 13D) in the BLA. Post hoc analysis with Dunn’s test indicated that NFC GW9662-treated rats had higher levels of dopamine levels in the right side compared to the left side (* *p* < 0.05). When each side was analysed separately, Kruskal–Wallis did not reveal a significant difference between groups in the right [χ^2^ (7) = 11.644, *p* = 0.053] and in the left [χ^2^ (7) = 8.987, *p* > 0.05] side. Because the right side almost reached statistical difference, an analysis considering the different fear conditioning groups was carried out. When we further analysed the fear conditioning groups, the Kruskal–Wallis test revealed a significant difference between groups in the NFC rats in the right [χ^2^ (3) = 8.324, *p* < 0.05] but not in the left [χ^2^ (3) = 5.168, *p* > 0.05] side. Post hoc analysis with Dunn’s test indicated that NFC rats treated with GW6471 had increased dopamine levels compared to the NFC Vehicle-treated ones (^#^ *p* < 0.05). The test also indicated a strong trend for increased levels of dopamine in the NFC GW9662-treated rats compared to the NFC vehicle-treated (*p* = 0.0584). The Kruskal–Wallis test did not reveal any significant differences between groups in FC rats neither in the right [χ^2^ (3) = 1.937, *p* > 0.05] nor in the left [χ^2^ (3) = 3.028, *p* > 0.05] side.

Kruskal–Wallis test also revealed a significant difference between groups (χ^2^ (15) = 25.622, *p* < 0.05) in the GABA levels (Figure 13A) in the BLA. However, post hoc analysis with Dunn’s test did not show any significant between-group differences in the GABA levels. When each side was analysed separately, Kruskal–Wallis did reveal a significant difference among groups in the right [χ^2^ (7) =14.483, *p* < 0.05] but not in the left [χ^2^ (7) = 3.012, *p* > 0.05] side. However, post hoc analysis with Dunn’s test did not show any significant between-group differences in the GABA levels.

Kruskal–Wallis test did not show any significant difference between groups in glutamate (χ^2^ (15) = 15.856, *p* > 0.05; Figure 13B) or serotonin (χ^2^ (15) = 22.532, *p* > 0.05; Figure 13C) levels in the BLA. When each side was analysed separately, Kruskal–Wallis did not reveal a significant difference in the levels of glutamate among groups in the right [χ^2^ (7) = 6.458, *p* > 0.05] or in the left [χ^2^ (7) = 3.802, *p* > 0.05] side. Similarly, when each side was analysed separately, Kruskal–Wallis did not reveal a significant difference in serotonin levels among groups in the right [χ^2^ (7) = 12.250, *p* > 0.05] or in the left [χ^2^ (7) = 2.039, *p* > 0.05] side.

#### 2.2.5. Effect of Fear Conditioning and PPAR Antagonist Administration on Endocannabinoid and NAE Levels in the Basolateral Amygdala (BLA)

Again, we investigated endogenous ligands for PPARs (AEA, PEA, and OEA) in the BLA, and checked for possible effects of PPAR antagonism in the absence of nociceptive tone on their levels.

**Figure 14:**
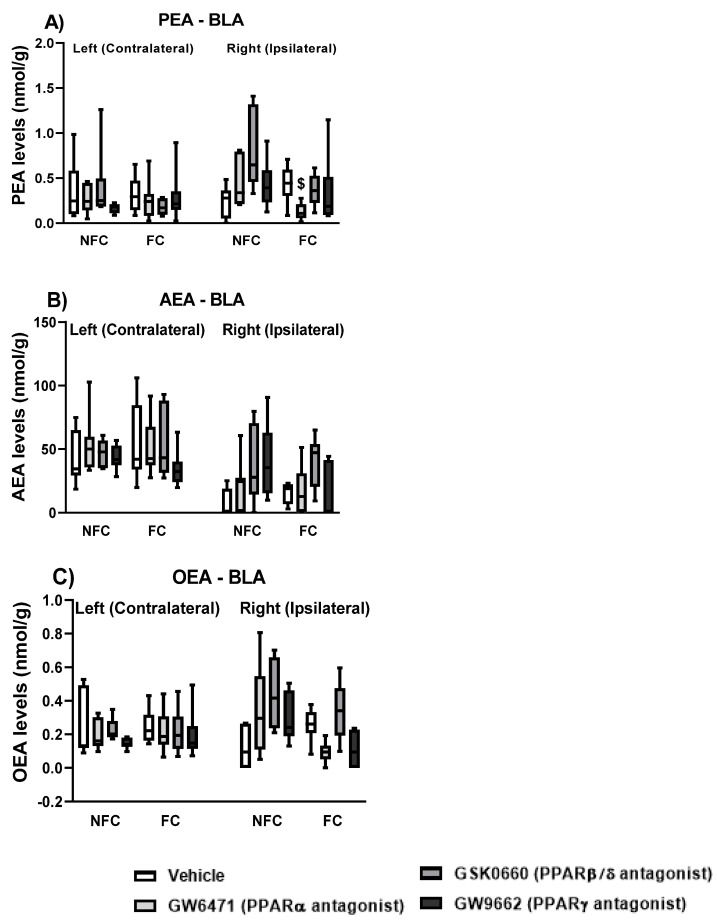
Effects of fear-conditioning and intra-BLA administration of selective PPARα, PPARβ/δ, and PPARγ antagonists on the levels of, PEA (**A**), AEA (**B**), and OEA (**C**). Post hoc analysis with Dunn’s test indicated that FC GW6471-treated rats had decreased levels of PEA compared to the FC Vehicle treated rats in the right BLA (^$^ *p* < 0.05). Data are expressed as median with interquartile range and min/max (*n* = 6–9 rats per group).

Kruskal–Wallis comparisons revealed a significant difference between groups (χ^2^ (15) = 32.124, *p* < 0.05) in the PEA levels (Figure 14A) in the BLA. However, post hoc analysis with Dunn’s test did not show any significant changes in PEA levels. When each side was analysed separately, two-way ANOVA revealed a significant effect of treatment [F (3, 44) = 3.034, *p* < 0.05], fear conditioning [F (1, 44) = 7.163, *p* < 0.05), and the interaction of treatment × fear conditioning [F (3, 44) = 3.606, *p* < 0.05] on the PEA levels in the right BLA. Post hoc analysis with Dunn’s test indicated that the FC GW6471-treated rats had decreased levels of PEA compared to the FC Vehicle treated rats in the right BLA (^$^ *p* < 0.05). Two-way ANOVA did not reveal any significant effect of treatment [F (3, 48) = 0.624, *p* > 0.05], fear conditioning [F (1, 48) = 0.590, *p* < 0.05], and the interaction of treatment × fear conditioning [F (3, 48) = 0.830, *p* < 0.05] on the PEA levels in the left BLA.

Kruskal–Wallis comparisons revealed a significant difference between groups (χ^2^ (15) = 47.410, *p* < 0.05) in the AEA levels (Figure 14B) in the BLA. However, post hoc analysis with Dunn’s test did not show any significant changes in the AEA levels. When each side was analysed separately, Kruskal–Wallis revealed a significant difference between the group in the right [χ^2^ (7) =14.798, *p* < 0.05] but not in the left [χ^2^ (7) = 6.537, *p* > 0.05] side. However, post hoc analysis with Dunn’s test did not show any significant changes in the AEA levels in the Right BLA.

Kruskal–Wallis comparisons revealed a significant difference between groups (χ^2^ (15) = 32.456, *p* < 0.05) in the OEA levels (Figure 14C) in the BLA. However, post hoc analysis with Dunn’s test did not show any significant changes in the OEA levels. When each side was analysed separately, Kruskal–Wallis test revealed a significant difference between groups in the OEA levels in the right (χ^2^ (7) = 21.988, *p* < 0.01) but not in the left (χ^2^ (7) = 6.350, *p* > 0.05) BLA. Post hoc analysis with Dunn’s did not reveal any significant differences between groups in the OEA levels in the right BLA.

### 2.3. Expression of PPARs in the BLA

#### 2.3.1. Western Blotting

**Figure 15:**
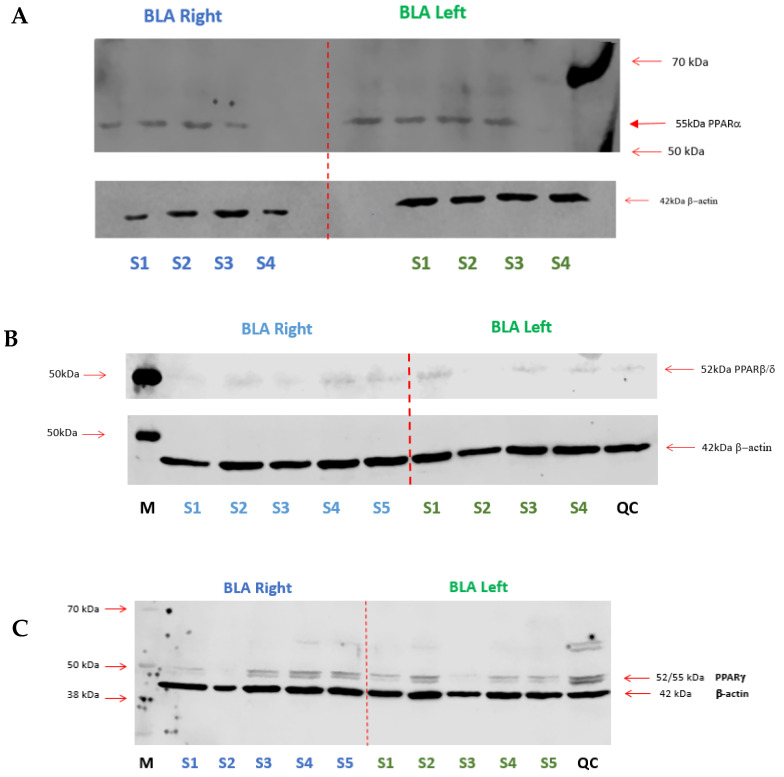
Expression of PPARα, PPARβ/δ, and PPARγ in the right and left BLA in four or five samples (S1–S4/S5 per side). The expression of PPARα (**A**) as seen at 55 kDa, PPARβ/δ (**B**) at 52 kDa, and PPARγ (**C**) at 52/55 kDa; β-actin was used as the endogenous control. M = marker/ladder; QC = quality control.

PPARα, PPARβ/δ, and PPARγ expression was confirmed in the right and left BLA of naïve male SD rats (Figure 15). The bands for PPARα (55 kDa) and PPARβ/δ (52 kDa) were obtained with the use of a monoclonal antibody. The double bands for PPARγ are a consequence of the expression of two subtypes of PPARγ: PPARγ_1_ and PPARγ_2_. The 42 kDa band corresponded to β-actin, used as an endogenous control.

#### 2.3.2. RT-qPCR

**Figure 16:**
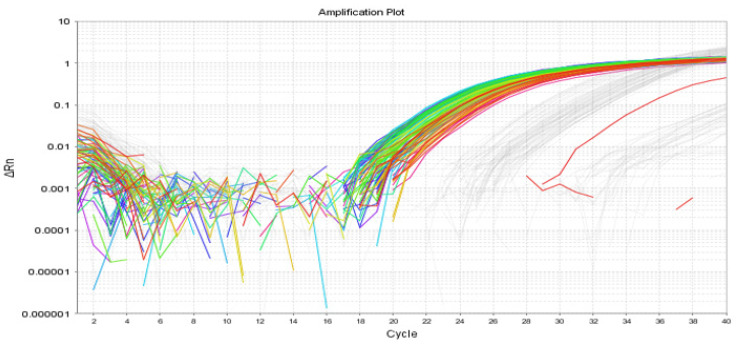
Amplification plots for PPARβ/δ gene expression in the right and left BLA.

The available antibodies developed to bind to PPARβ/δ in western blotting protocols did not provide results that were entirely satisfactory, as evidenced by the faint bands above (Figure 15B). Therefore, we opted to demonstrate the presence of PPARβ/δ in the BLA using RT-qPCR. The presence of mRNA encoding PPARβ/δ was confirmed in the right and left BLA of naïve male SD rats. The Ct values found for the BLA punches were 30.05 ± 0.11 in the BLA Right and 29.74 ± 0.02 in the BLA Left. Data are expressed as means ± S.D. (Figure 16)

### 2.4. Effects of Intraplantar Administration of Formalin on Levels of Neurotransmitters, Endocannabinoids, and NAEs in NFC and FC Rats

Samples from the NFC and FC vehicle-treated groups from both experiments were re-run on LC-MS/MS and re-analysed together in order to compare the possible effects of the presence of a nociceptive inflammatory tone (i.e., formalin) *versus* its absence (i.e., saline) on the levels of neurotransmitters, endocannabinoids, and NAEs.

#### 2.4.1. Effects of Intraplantar (i.pl.) Administration of Formalin on Levels of Neurotransmitters in NFC and FC Rats

**Figure 17:**
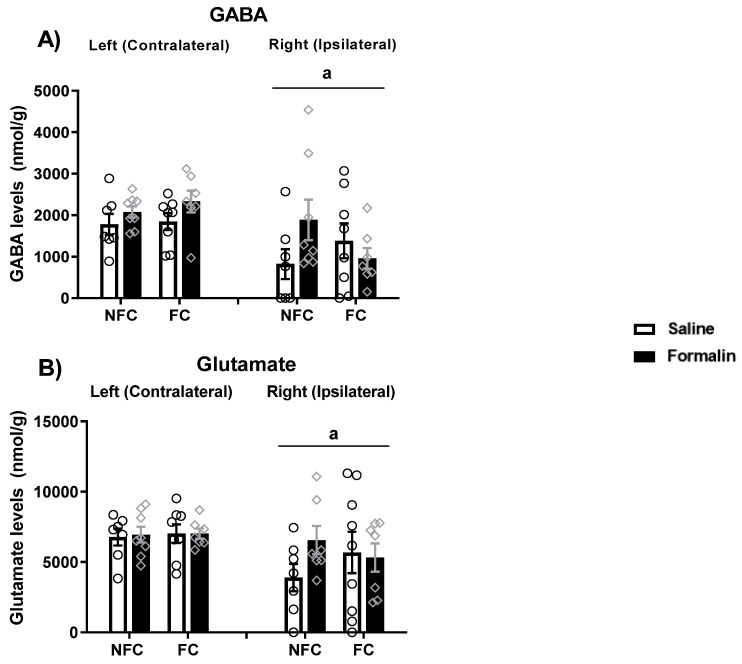

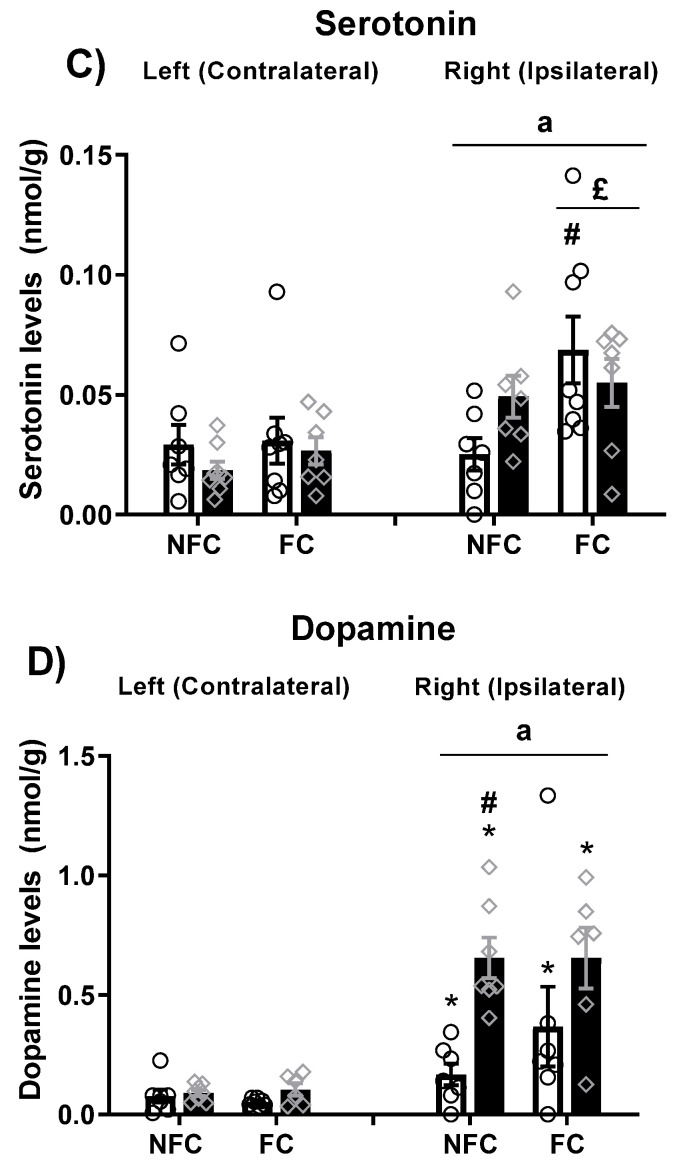
Effects of fear-conditioning and intraplantar injection of formalin on the levels of GABA (**A**), glutamate (**B**), serotonin (**C**), and dopamine (**D**). Two-way ANOVA revealed a significant effect of side on all neurotransmitters (^a^ *p* < 0.05). Post hoc pairwise analysis with Student Newman–Keuls showed a significant difference in serotonin levels between the NFC Vehicle and FC Vehicle of i.pl. saline-treated rats (^#^ *p* < 0.05), and dopamine levels between the NFC Vehicle i.pl. saline-treated and NFC Vehicle i.pl. formalin-treated rats (^#^ *p* < 0.05). The test also confirmed side differences (* *p* < 0.05, compared to their left counterparts) in the dopamine levels. Data are expressed as mean ± S.E.M, each symbol represents one individual, (*n* = 7–9 rats per group).

There was a significant effect of side (ipsilateral/contralateral) for all four neurotransmitters analysed (GABA [F (1, 52) = 10.730, ^a^ *p* = 0.002]; glutamate [F (1, 52) = 5.630, ^a^ *p* = 0.021]; serotonin [F (1, 51) = 12.192, ^a^ *p* = 0.001]; and dopamine [F (1, 47) = 53.882, ^a^ *p* < 0.001]; Figure 17). Post hoc pairwise analysis with Student Newman–Keuls did not show any further significant statistical differences for GABA and glutamate (Figure 17A,B), but indicated that saline-treated FC rats had increased levels of serotonin compared to their NFC counterparts (NFC Saline-treated vs. FC Saline-treated, ^#^ *p* < 0.05; Figure 17C) on the right side and also showed that NFC rats that received an intraplantar injection of formalin had increased levels of dopamine on the right BLA (NFC saline-treated vs. NFC-formalin-treated, ^#^ *p* < 0.05; Figure 17D). It also confirmed the side differences (* *p* < 0.05, compared to their left counterparts) in the dopamine levels (Figure 17D). When the right and left sides were analysed separately, two-way ANOVA revealed a significant effect of fear conditioning [F (1, 24) = 4.464, ^£^ *p* < 0.05] on the serotonin levels in the right BLA. However, post hoc pairwise analysis with Student Newman–Keuls did not show significant statistical differences. The other comparisons did not reach any statistical significance (see the Supplementary Materials for details).

#### 2.4.2. Effects of Intraplantar Administration of Formalin on Levels of Endocannabinoids and NAEs in NFC and FC Rats

**Figure 18:**
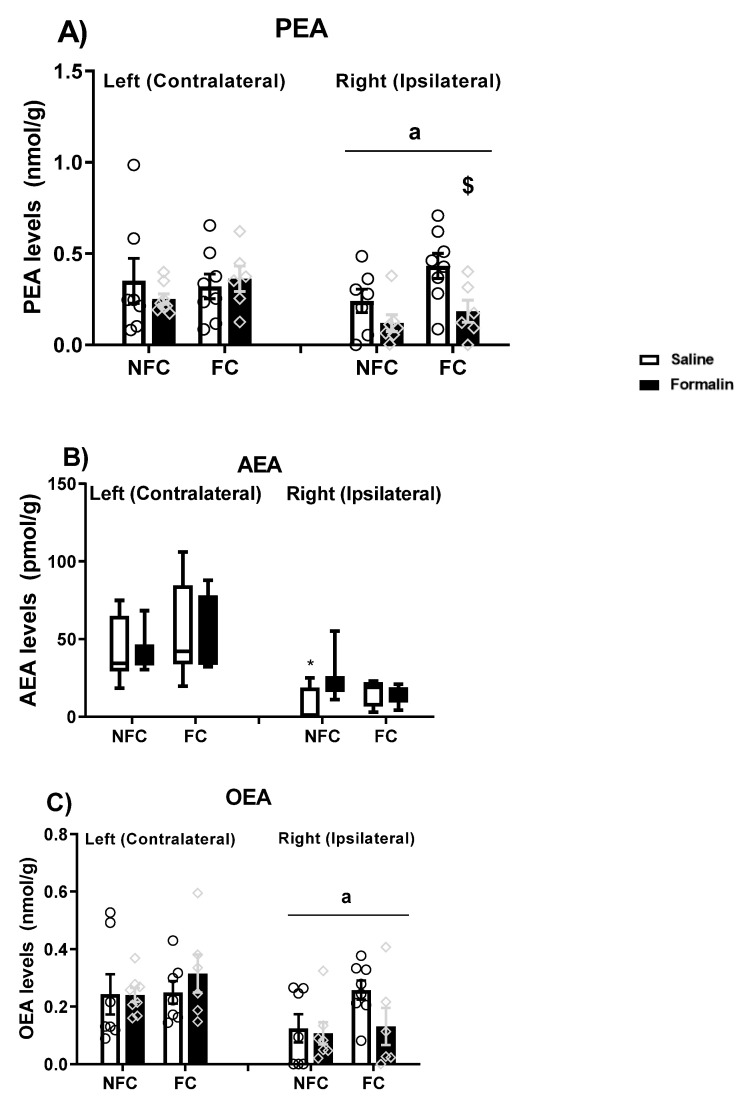
Effects of fear-conditioning and intraplantar injection of formalin on the levels of PEA (**A**), AEA (**B**), and OEA (**C**). Two-way ANOVA revealed a significant effect of side on PEA and OEA (^a^ *p* < 0.05). Post hoc pairwise analysis with Student Newman–Keuls indicated that FC rats that received formalin injection had lower levels of PEA in the right side compared to their saline-treated counterparts (FC formalin-treated vs. FC saline-treated, ^$^ *p* < 0.05). Data are expressed as mean ± S.E.M (**A**,**C**) or median with interquartile range (**B**); each symbol represent one individual, *n* = 6–9 rats per group.

Likewise, an effect of side (ipsilateral/contralateral) was seen for two of the endogenous ligands at the PPARs analysed (PEA: [F (1, 49) = 4.191, ^a^ *p* = 0.046]; OEA: [F (1, 48) = 9.699, ^a^ *p* = 0.003]; Figure 18A,C). The following post hoc pairwise analysis with Student Newman–Keuls did not show significant statistical differences in PEA and OEA levels. When the right and left sides were analysed separately, two-way ANOVA revealed a significant effect of treatment [F (1, 23) = 8.216, *p* = 0.009] on the PEA levels in the right BLA (Figure 18A). Post hoc pairwise analysis with Student Newman–Keuls indicated that FC rats that received formalin injection had lower levels of PEA in the right side compared to their saline-treated counterparts (FC formalin-treated vs. FC saline-treated, ^$^ *p* < 0.05). There were no significant differences in the levels of OEA when sides were analysed separately (Figure 18C; see the Supplementary Materials for details).

Kruskal–Wallis comparisons revealed a significant difference between groups (χ^2^ (7) = 35.131, *p* < 0.05) in the AEA levels (Figure 18B). Post hoc analysis with Dunn’s test showed lower levels of AEA in the NFC Saline group in the right side compared to the left (* *p* < 0.05). When each side was analysed separately, Kruskal–Wallis did not reveal any significant differences between groups in the right [χ^2^ (3) =6.485, *p* > 0.05] or in the left [χ^2^ (3) = 2.456, *p* > 0.05] side.

## 3. Discussion

The experiments described herein investigated the role of PPARs expressed in the BLA in the mediation or modulation of inflammatory pain, FCA, and conditioned fear, the latter in the presence and absence of nociceptive tone. The expression of the three isoforms in the BLA was confirmed by western blotting (and re-confirmed by RT-qPCR in the case of PPARβ/δ). Administration of GW6471, a PPARα antagonist, directly into the BLA prolonged freezing duration in FC rats in the presence of formalin-evoked nociceptive tone and increased freezing duration in NFC rats in the absence of nociceptive tone. The administration of a PPARγ antagonist, GW9662, into the BLA enhanced freezing expression in the first part of the trial in the presence, but not in the absence, of nociceptive tone. Thus, endogenous PPAR signalling through PPARγ expressed in the BLA may act to attenuate or extinguish conditioned fear behaviour. Likewise, PPAR signalling through PPARα expressed in the BLA seems to be involved in the recall of fear-related memories, with its blockade resulting in potentiation of fear conditioned behaviour in the first part of the trial. Importantly, these effects were only seen in the presence of formalin-evoked nociceptive tone; they were not observed in rats that received intraplantar saline injection. The effects of PPARα and PPARγ blockade on freezing expression were associated with increased levels of dopamine in the right BLA. In the absence of nociceptive tone, the administration of the three antagonists increased freezing duration in NFC rats. These results suggest a modulatory role for PPARs in innate anxiety, but not in conditioned fear, in the absence of nociceptive tone. The intra-BLA injection of PPAR antagonists did not alter nociceptive behaviour or locomotor activity in either NFC or FC rats, irrespective of the nociceptive status. These results suggest that PPAR signalling in the BLA does not modulate pain or FCA. Taken together, these results demonstrate a differential effect of the PPAR signalling system on fear and/or anxiety in the presence versus absence of acute inflammatory pain.

Extinction is defined as a learned inhibition of the retrieval of previously acquired memories. Many studies have demonstrated that PPAR signalling plays a role in mnemonic formation [48,49,50,51]. However, the role of PPARs expressed in the BLA in memory and learning formation have not been investigated yet. We propose that the blockade of PPARα expressed in the BLA delayed short-term, within-trial extinction of fear memory in the presence of nociceptive tone. The blockade of PPARγ in the same region potentiated the initial freezing expression, but did not affect its extinction, in the presence of a nociceptive tone. These effects are related to increased levels of dopamine in the right BLA of FC rats, both in the presence and absence of nociceptive tone, suggesting a possible link of PPAR signalling and basolateral amygdalar dopaminergic modulation of fear and anxiety responses. Our findings are in agreement with a recent study showing that PPARα-KO mice had enhanced fear learning compared to their WT counterparts, and that this enhancement is associated with increased levels of dopamine in the amygdala [51]. Other studies have proposed that PPARs modulate dopamine signalling. Mijangos-Moreno et al. (2016) [52] showed that WY14643 (PPARα agonist) injected into the hypothalamus increased dopamine levels in the nucleus accumbens. This same agonist and methOEA (a long lasting form of OEA), when systemically administered, dose-dependently decreased nicotine-induced excitation of dopamine neurons in the VTA and nicotine-induced elevations of dopamine levels in the nucleus accumbens shell of rats [53]. Thus, we hypothesize that the blockade of PPARα in the BLA of FC rats affects dopamine signalling within this region, resulting in a delay in extinction learning. Moreover, the blockade of PPARγ in FC rats affects dopamine signalling in the BLA, which in turn could result in the enhancement of the recall of fearful memories [54]. Alternatively, the blockade of these receptors may have affected AEA action on fear expression and/or extinction. Previous work from our group has shown increased levels of AEA in the BLA of FC rats that received intraplantar formalin injection in the hind paw compared to NFC counterparts, and trends were also present for the other two NAEs—PEA and OEA [55]. Recently, Morena et al. (2018) [56] demonstrated that the overexpression of FAAH in the BLA decreased expression of conditioned fear in the extinction training session and anxiety-related behaviour in rats. We hypothesize that AEA in the BLA may modulate fear processing through PPARα and PPARγ. Thus, the blockade of these receptors may have affected AEA action on fear expression and/or extinction. However, a possible role of PEA and OEA in this modulation cannot be disregarded. Further studies focusing on the activation of PPARs and the role of PEA and OEA signalling in the BLA in conditioned fear and anxiety could contribute to a better understanding of the role of PPAR signalling in the BLA in conditioned fear and anxiety.

Recent studies have pointed to a possible role of PPARs in anxiety and cognition. Our recent work has shown that the administration of a PPARα antagonist exacerbated inflammatory pain-related impairment of spatial memory in rats [57]. Youssef et al. (2019) showed that the administration of a PPARγ antagonist blocked the anxiolytic effect of beta-caryophyllene. Another study demonstrated that repeated stress decreased PPARγ expression in the amygdala, and treatment with anxiolytics recovered PPARγ expression [58]. PPARγ blockade or knockout was shown to have anxiogenic effects on mice [43]. In this same study, intra-amygdala injections of pioglitazone (PPARγ agonist) were shown to reduce stress-induced anxiety behaviour in rats. In the present study, NFC rats that received intra-BLA injections of PPAR antagonists in the absence of nociceptive tone had increased levels of freezing, comparable to their FC counterparts. Thus, the blockade of these receptors in the BLA increased the innate anxious state in NFC rats with absent formalin-evoked pain. The studies to-date have investigated the role of PPARs in provoked anxious state (i.e., stress or pharmacological-induced anxiety state). Our results support and extend these studies, demonstrating that PPAR signalling in the BLA may modulate anxiety-related behaviour in the absence of nociceptive tone.

The results suggest that PPAR signalling in the BLA does not mediate or modulate formalin-evoked nociceptive behaviour. As previously mentioned, other studies have demonstrated effects of PPAR agonists on pain-related behaviour [59,60,61,62,63] but less is known about the effect of PPAR antagonists. The exogenous administration of PPAR natural ligands has also been shown to modulate pain responses (see Okine et al. 2018 for a review). To our knowledge, the present study is the first to investigate the effect of the blockade of PPARα, PPARβ/δ, and PPARγ expressed in the BLA on inflammatory pain. Similarly to what was shown by us previously [47] and by Donvito et al. (2017) and Mansouri et al. (2017) in their systemic studies, PPAR antagonist administration into the BLA did not affect formalin-evoked nociceptive behaviour.

FCA is a potent suppression of nociceptive responses upon exposure to a fearful stimulus. Our current study investigated the effects of intra-BLA administration of PPAR antagonists on FCA. FCA has been previously shown to be associated with increased levels of AEA, an endocannabinoid that also binds to PPARs, in the BLA [55] and a strong trend for increased tissue levels of PEA and OEA, endogenous ligands of PPARs, in the BLA. No FCA-related alterations in the levels of AEA, PEA, and OEA in the BLA were seen in our experiment; however, Rea et al. (2013) [64] used a different rat strain (Lister-hooded vs. Sprague-Dawley) and a shorter trial compared to our experiment (15 min vs. 30 min), which may explain the different observations in our studies. The data demonstrate that fear conditioning profoundly reduces formalin-evoked nociceptive behaviour via FCA as we and others have previously shown [65,66,67,68,69,70] and that the blockade of PPARα, PPARβ/δ, or PPARγ in the BLA does not affect the expression of FCA.

Furthermore, we compared the effects of intraplantar injection of formalin or saline on the tissue levels of neurotransmitters, endocannabinoids, and NAEs in the BLA of FC and NFC rats. The presence or absence of formalin-induced inflammatory pain was shown to influence the changes in the levels of neurotransmitters and NAEs after fear conditioning rats that received intra-BLA vehicle. For example, FC rats that received saline injection into the right hind paw had increased serotonin and AEA levels in the right BLA, but these effects were not observed in formalin-treated animals. Fear conditioning also decreased PEA levels in the right BLA of formalin-, but not saline-, treated rats. Rea et al. (2013) showed that PEA levels were higher in the left BLA of FC formalin compared to NFC formalin-treated counterparts, which is opposite to what we have observed in our experiment, in which PEA levels were higher in the right BLA of FC formalin compared to the NFC formalin-treated counterparts. In addition, Rea et al. (2013) did not observe altered AEA levels in the left or right BLA of saline-treated animals, contrary to what we observed in our study, where the AEA levels were higher in the FC saline-treated compared to NFC saline-treated rats. However, the rats used by Rea et al. (2013) were from a different strain (Lister-hooded versus Sprague Dawley), which may explain the difference in the results between our studies. The higher levels of serotonin in FC saline-treated rats compared to NFC counterparts that we have observed were in accordance to what Zanoveli et al. (2009) [71] observed in their microdialysis study. Similarly, serotonin levels were increased in conditioned rats 30 min after re-exposure. Other studies have also shown that serotonin neurotransmission in the BLA can be involved in the facilitation of conditioned states [72,73,74]. Formalin-injection itself affected dopamine and AEA levels. NFC rats that received an intraplantar formalin injection were shown to have increased levels of both dopamine and AEA in the right BLA compared to their saline-treated counterparts. Although the role of dopaminergic signalling in pain responses has been extensively studied [75,76,77,78], the role of this system in the amygdala in nociception has been less examined. Roche et al. (2007) [79] did not find changes in dopamine levels in the amygdaloid complex of formalin-treated rats compared to saline-treated rats, which is in contrast with our findings. The rats, similar to our study, underwent cannula implantation into the BLA but were only re-exposed for 15 min to the arena. Additionally, the levels of dopamine were measured using a different technique (i.e., HPLC with electrochemical detection) in gross dissected amygdala in contrast to punches of each of the amygdalar nuclei separately. One study demonstrated that antagonism of D_1_ receptors in the BNST (part of the extended amygdala) enhanced nociceptive responses in female, but not male rats, suggesting that the dopaminergic system in the BNST may exert sexually dimorphic effects on pain [80]. The blockade of dopaminergic receptors in the nucleus accumbens prevented antinociceptive effects of CB_1_ receptor activation in the BLA, suggesting a link between the neuromodulation of pain in the BLA and the mesolimbic dopaminergic system. The blockade of D_2_ and D_4_ in the PFC inhibited long lasting suppression of nociceptive responses induced by high frequency stimulations of the BLA, suggesting a link between the neuromodulation of pain and the prefrontal dopaminergic system. In their investigation, Rea et al. (2013) did not see any changes in AEA levels in the BLA of formalin-treated rats compared to saline-treated counterparts, which is divergent to what we observed in our results. However, their re-exposure time to the conditioning arena was longer (45 or 60 min) than the one used in our experiments (30 min), which may account for this difference. In our experiment, FC formalin-treated rats had higher levels of PEA in the right BLA compared to saline-treated animals, a result also seen by Rea et al. (2013). Together, these results show that intraplantar formalin injection impacts neurotransmitters and NAE signalling in the BLA. Thus, it is possible that these neurochemical differences underpin the differential effects of PPAR blockade on conditioned fear-related behaviour in the presence versus absence of formalin-evoked nociceptive tone.

In conclusion, the experiments described herein have shown that the blockade of PPARα expressed in the BLA impaired short-term, within trial fear-extinction, and the blockade of PPARγ in the same region potentiated freezing expression in the presence of a nociceptive stimulus in rats, without affecting pain responses. Moreover, the blockade of PPARα, PPARβ/δ, and PPARγ in the BLA increased innate anxiety status in the absence of pain in NFC rats. These results indicate a possible modulatory role for PPARs in the BLA in fear/anxiety expression, with differential effects depending on the presence or absence of nociceptive tone. Further investigations are necessary to elucidate the possible mechanisms involved in these modulations and clarify the molecular basis of this differential pain-dependent effect.

## 4. Materials and Methods

### 4.1. Animals

Experiments were carried out on a total of 88 (Experiment 1) and 92 (Experiment 2) adult male Sprague-Dawley rats (230–250 g on arrival; Envigo UK, Bicester, England). The animals were maintained at a controlled temperature (22 ± 2 °C) and humidity (45–55%) under standard lighting conditions (12:12 h light–dark cycles, lights on from 07.00 h). All experiments were carried out during the light phase. Food and water were available ad libitum. The experimental procedures were approved by the Animal Care and Research Ethics Committee, National University of Ireland Galway. The work was carried out under license from the Health Products Regulatory Authority in the Republic of Ireland and in accordance with EU Directive 2010/63.

### 4.2. Cannula Implantation

Under isoflurane anaesthesia (2–3% in O_2_, 0.7 L/min), a stainless steel guide cannula (12 mm length, Plastics One Inc., Roanoke, VA, USA) was stereotaxically implanted 1 mm above the right and left BLA of each rat (coordinates: AP = −2.5 mm from bregma, ML = ±4.8 mm, DV = −7.5 mm from the skull surface) according to the rat brain atlas published by Paxinos and Watson (Paxinos et al. 1997) [81]. The cannulae were permanently fixed to the skull using stainless steel screws and carboxylate cement. A stylet made from stainless steel tubing (12 mm length, 22 G, Plastic One—Bilaney Consultants, Sevenoaks, UK) was inserted into the guide cannula to prevent blockage by debris. The non-steroidal anti-inflammatory agent, carprofen (1.25 mg/25 µL, s.c., Rimadyl, Pfizer, Kent, UK), was administered before surgery to manage postoperative analgesia. Animals received a single daily dose of the antimicrobial agent enrofloxacin (10 mg/kg, s.c., Baytril, Bayer plc, Berkshire, UK) for five days to prevent postoperative infection. Following cannula implantation, the rats were singly housed and at least seven days were allowed for recovery post-surgery prior to experimentation. During this recovery period, the rats were handled, stylets checked, and their body weight and general health monitored once daily.

### 4.3. Drugs

PPARα antagonist, GW6471, PPARβ/δ antagonist, GSK0660, and PPARγ antagonist, GW9662 (all obtained from Tocris Bioscience, Bristol, UK) were dissolved in 100% dimethyl sulfoxide (DMSO), which was used as a vehicle solution. The dose of GW6471 (10 ng/0.5 μL) was chosen based on a study from our laboratory showing that this dose delayed the onset of the second phase of formalin-evoked nociceptive behaviour [43]. The dose of GW9662 (10 ng/0.5 μL) was chosen based on a previous study showing that this dose was effective in reversing the anti-inflammatory and anti-hyperalgesic actions of rosiglitazone [82]. We used the same dose of GSK0660 (10 ng/0.5 μL) as that used for the other two antagonists for comparison and because, up to the time of the experiments, no published studies have administered this drug intracerebrally. Formalin was prepared from a 37% stock solution (Sigma-Aldrich, Dublin, Ireland) diluted in sterile saline. Sodium chloride was dissolved in distilled water (9 g in 1 L—0.9%) and the solution was autoclaved.

### 4.4. Experimental Procedure

Two different experiments using two different cohorts of rats were carried out (Experiments 1 and 2) and identical in design and methodology with the exception that rats in Experiment 1 received an intraplantar injection of formalin while those in Experiment 2 received an intraplantar injection of saline. The FCA paradigm used in both experiments was essentially as previously described [83,84,85]. There were two phases: conditioning (day 1) and testing (day 2). On the conditioning day, rats were placed in a Perspex chamber (30 cm × 30 cm × 40 cm) and after 15 s, they received the first of 10 footshocks (0.4 mA, 1 s duration, LE85XCT Programmer and Scrambled Shock Generator; Linton Instrumentation, Norfolk, UK) spaced 60 s apart. Fifteen seconds after the last footshock, rats were returned to their home cage. The animals that belonged to the control group, which did not receive footshocks, were placed in the chamber for an equivalent time (9 min 30 s). The animals were randomly assigned to one of eight groups (*n* = 11 per group; see Supplementary Table S1 for a summary of experimental groups)—rats that received footshock (FC) or no footshock (NFC) treated with the PPARα antagonist GW6471, PPARβ/δ antagonist GSK0660, PPARγ GW9662, or vehicle (100% DMSO). The sequence of testing was randomised to minimise any confounding effects of the order of testing.

**Figure 19:**
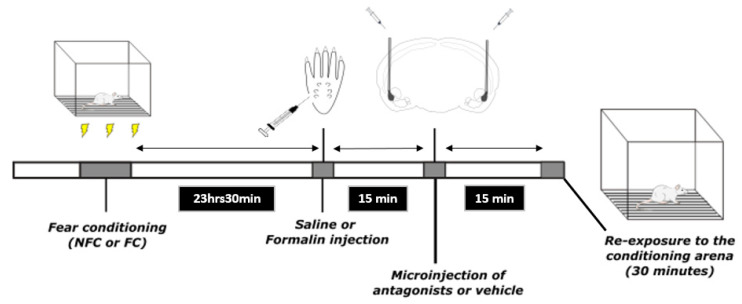
Graphical representation of the experimental procedure.

The test day started 23 h 30 min after the end of the conditioning phase (Figure 19). First, the rats received a 50 µL injection of formalin (2.5% in saline; Experiment 1) or saline (Experiment 2) into the right hind paw under brief isoflurane anaesthesia (3% in O_2_; 0.8 L·min^−1^). Fifteen minutes after, the animals received intra-basolateral amygdalar (intra-BLA) microinjections of either the PPARα antagonist (GW6471), the PPARβ/δ antagonist (GSK0660), PPARγ antagonist (GW9662), or vehicle (volume of injection 0.5 µL/side). After these microinjections, the rats were returned to their home cages. Fifteen minutes after the microinjections, or 24 h after footshock, the rats were re-exposed to the conditioning chamber. A video camera located beneath the observation chamber was used to monitor animal behaviour for 30 min. At the end of the test phase (60 min post formalin injection), rats were killed by decapitation, fast-green dye injected via the guide cannulae, brains were removed, snap-frozen on dry ice, and stored at −80 °C. Formalin induced oedema was assessed by measuring the change in the diameter of the right hind paw measured immediately before, and 60 min after, formalin administration, using Vernier callipers.

### 4.5. Behavioural Analysis

Behaviour was analysed using the Ethovision 11.5 XT software package (Noldus Technology, Wageningen, The Netherlands). A trained observer blind to the experimental conditions assessed behaviour including: (1) freezing duration (defined as the absence of visible movement except that needed for respiration); (2) duration of walking; (3) duration of grooming; and (4) duration of rearing. Moreover, formalin-evoked nociceptive behaviour was scored according to the composite pain scoring (CPS) technique described by Watson et al. (1997) [86] in which pain behaviours are classified as time spent raising the formalin-injected paw (P1), and holding, licking, biting, shaking, or flinching the injected paw (P2). Thus, we obtained a CPS value from the equation [CPS = (P1 + 2(P2))/(total duration of trial)].

### 4.6. Histological Verification of Intracerebral Injection Sites

Stereotaxic coordinates were verified histologically on two animals before the start of the cannula implantation surgeries. The rats underwent the surgical procedure as previously described. After the conclusion of the surgical implantation of cannulae, the two rats, still under anaesthesia, were decapitated and a microinjection of 2% fast green dye (0.5 μL over 1 min; Sigma-Aldrich, Dublin, Ireland) diluted in DMSO was made to determine whether the coordinates used were accurate for the BLA. The brain was collected and snap-frozen on dry ice. Then, frozen coronal brain sections were cut at a 50 μm thickness on a cryostat at −21 °C from the start to the end of the amygdaloid complex to determine the location of the dye and confirm the coordinates. For all other rats in the experiments, the dye injections were performed immediately post-decapitation in order to determine if the injections successfully targeted the BLA.

### 4.7. Cryo-Sectioning and Tissue Microdissection

Frozen coronal brain sections of 150 µm thickness containing the basolateral amygdala (BLA) were cut on a cryostat (Leica Biosystems, Watznal, Germany) and punch-dissected as previously described [70,87,88] using cylindrical brain punchers (Harvard Apparatus, Holliston, MA, USA) with an internal diameter of 0.50 mm for the different amygdalar nuclei, at the following rostro-caudal levels: Bregma, −2.12–−3.30 mm. Additionally, in order to evaluate possible lateralisation effects, the BLA punches were separately collected for the right and left hemispheres. The punch-dissected tissue was weighed (mean ± S.E.M. weight per sample was 1.72 ± 0.1 mg) and stored at −80 °C prior to measurement of the AEA, PEA, OEA, 2-AG, and neurotransmitter levels by liquid chromatography coupled to tandem mass spectrometry (LC-MS/MS).

### 4.8. Measurement of Endocannabinoids, NAEs, and Neurotransmitters in Discrete Brain Regions Using Liquid Chromatography—Tandem Mass Spectrometry (LC-MS/MS)

Each punch-dissected brain sample was homogenised for 4–6 s with an ultrasonic homogeniser/sonicator (Mason, Dublin, Ireland) in a mixture containing 200 μL of deuterated internal standards for endocannabinoids (0.48 nmol/50 ng of 2-AG-d8 and 0.014 nmol/2.5 ng of AEA-d8) and NAEs (0.015 nmol/2.5 ng of OEA-d2 and 0.016 nmol/2.5 ng of PEA-d4), and 10 μL of deuterated internal standards for neurotransmitters (5 μg/0.048 μmol of GABA-d6, 5 μg/0.033 μmol of glutamate d-5, 1 ng/0.006 nmol of dopamine-d-4, and 1 ng/0.005 nmol of serotonin-d-4) and immediately kept on ice. The final volume was made up to 260 μL prior to sonication by adding 50 μL of 100% acetonitrile. Deuterated and non-deuterated endocannabinoids were purchased from Cayman Chemicals (Biosciences, Cambridge, UK). Non-deuterated neurotransmitters were purchased from Sigma Chemicals (Dublin, Ireland): 2129-GABA, G1251-glutamate, H8502-dopamine, and H9523-serotonin. Deuterated neurotransmitters for GABA, glutamate, and dopamine were acquired from CDN isotopes (Pointe-Claire, QC, Canada) (D1828-GABA (D6), D2193-glutamate (D5), D1540-dopamine (D4)). The deuterated serotonin was procured from Alsachim (Strasbourg, France) M760-serotonin (D4).

Samples were kept on ice during the procedure. The homogenates were centrifuged at 11,000× *g* for 15 min at 4 °C (Hettich centrifuge Mikro 22R, Steinheim am Albuch, Germany). The supernatant was collected and 40 μL was transferred to a HPLC vial. The standard curve was constructed using serial 1/2 dilution by adding 50 µL of a mixture of non-deuterated endocannabinoids and NAEs (25 ng for PEA, OEA, and AEA + 250 ng for 2-AG) and 10 µL of a mixture of non-deuterated neurotransmitters (100 µg of glutamate and GABA, 10 ng each of dopamine, noradrenaline, and serotonin) to 40 μL of acetonitrile in tube #10, vortex-mixing, then collecting 50 µL and transferring it to the next tube (#9) containing 50 µL acetonitrile. The process was repeated until tube #1, when 50 µL of the final volume was discarded, in order to keep the volumes between tubes consistent. Thus, all 10 tubes had 50 µL of a mixture of endocannabinoids and neurotransmitters. All standard curve tubes were spiked with 200 µL of a deuterated endocannabinoid/NAE mixture (2.5 ng deuterated PEA, OEA, and AEA and 50 ng deuterated 2-AG as the internal standards) and 10 µL of deuterated neurotransmitter mixture (5 µg of glutamate and GABA, and 1 ng each of dopamine, and serotonin). A double blank (100% acetonitrile) was also included in between each standard point during the run to minimise the risk of analyte carryover from standard to standard at the upper range of the curve and five double blanks were included after the highest concentration point on the curve to avoid carryover onto the samples. A quality control (QC) sample was prepared from the whole rat brain homogenate using the same protocol described for the punches, and was included with each run to allow for monitoring of inter-run variability. The QC was added after all the samples, at the end of the run.

Mobile phases consisted of (1) high pressure liquid chromatography (HPLC) grade water with 0.1% (*v*/*v*) formic acid, and (2) acetonitrile with 0.1% (*v*/*v*) formic acid for the initial three minutes with a flow rate of 0.2 mL/min using a Waters Atlantis T3 column (3 µm particles, 100 mm length, 2.1 mm diameter; Waters, UK). Quantitation of each analyte was performed by determining the peak area response of each target analyte against its corresponding deuterated internal standard. This ratiometric analysis was calculated using Masshunter Quantitative Analysis Software (Agilent Technologies Ltd., Cork, Ireland). The amount of analyte in unknown samples was calculated from the analyte/internal standard peak area response ratio using a 10-point calibration curve constructed from a range of concentrations of the non-deuterated form of each analyte and a fixed amount of deuterated internal standard. The values obtained from the Masshunter Quantitative Analysis Software are initially expressed in ng per mg of tissue by dividing by the weight of the punched tissue. To express values as nmol or pmols per mg, the corresponding values are then divided by the molar mass of each analyte expressed as ng/nmole or pg/pmole.

### 4.9. Verification of PPAR Expression in the BLA

Previous studies showing PPAR expression in the amygdalar complex [41,42] had not considered the subdivisions of the amygdala or all the isoforms in their experiments. Therefore, we carried out a confirmation of the expression of each of the isoforms of PPARs in the BLA.

#### 4.9.1. Verification of PPAR Expression in the BLA by Western Blotting

Punched brain tissues from BLA of naïve male SD rats were analysed by western immunoblotting. Frozen punched samples were lysed briefly with 3 s sonication in radio-immunoprecipitation assay (RIPA) lysis buffer (150 mmol/L NaCl, 25 mmol/L Tris-HCl, pH 7.6, 0.5% Triton X-100, 1% sodium deoxycholate, 0.1% sodium dodecyl sulphate, 1 mmol/L Na_3_VO_4_, 10 mmol/L NaF containing 1% protease inhibitor cocktail [Sigma-Aldrich, Ireland] in a 1.5 mL microcentrifuge tube [75 mL]). After homogenisation, the microcentrifuge tube was placed on the shaker for 45 min at 4 °C for the RIPA lysis buffer to free the protein bound either to the plasma membrane/nuclear membrane and then centrifuged at 14,000× *g* (Eppendorf Centrifuge 5415R, Stevenage, UK) for 20 min at 4 °C to separate the precipitate and the supernatant. The supernatant was collected, and the protein content determined by the Bradford assay. Protein (BSA, Sigma-Aldrich, Arklow, Ireland) standards (0, 0.0125, 0.25, 0.5, 0.75, 1.0, 1.5, 2.0 mg/mL) were prepared in deionised water (DH_2_O). The Bradford assay involved adding 250 μL of Bradford reagent (Sigma-Aldrich, Ireland) to 5 μL of unknown samples or standards in triplicate on a 96-well plate. After a 5 min incubation time, absorption at 570 nm wavelength was determined. Protein concentrations of the samples were determined using an 8-point standard curve constructed using the BSA standards. The samples were equalised to 2.0 mg/mL after determining the protein concentration. Eight μL of 4X sample loading buffer was added to 24 μL of the protein sample (48 μg of protein sample) in the microcentrifuge tubes (4X sample loading buffer: 25% *v*/*v* 1 mol/L Tris-HCl, pH 6.8, 5% *w*/*v* sodium dodecyl sulphate (SDS), 20% *v*/*v* glycerol, 2.5% bromophenol blue (0.2% *w*/*v* in 100% ethanol), 7 M urea, and 20% *v*/*v* of 2-mercaptoethanol, made up to a total volume of 20 mL in distilled water). The microcentrifuge tubes were vortexed quickly and then boiled at 95 °C for 5 min. The samples were then briefly centrifuged and subjected to 9% SDS–polyacrylamide gel electrophoresis (SDS-PAGE) at a constant voltage of 120 mV for 2 h. The separated protein samples were electroblotted onto a nitrocellulose membrane (Nitrocellulose membrane, CAS# 9004-70-0; Bio-Rad, Hercules, CA, USA) at 100 mV for 40 min using the wet transfer method. Protein transfer efficiency was verified by ponceau S (0.1% ponceau dye in 5% acetic acid; Sigma-Aldrich, Arklow, Ireland) staining of the protein band. Membranes were blocked in 5% non-fat dry milk in 0.1% Tris-buffered saline/Tween 20 (TBST) solution for 1 h at room temperature and incubated with a polyclonal antibody to PPARβ/δ Cat# 398394, anti-rabbit, Santa Cruz Biotechnology, Dallas, TX, USA], PPARα [1:200, Cat# 74517, anti-mouse Santa Cruz Biotechnology, Dallas, TX, USA] or PPARγ receptor [1:200, Cat# 22020, anti-goat, Santa Cruz Biotechnology, Dallas, TX, USA] and mouse monoclonal antibody to β-actin (1:10000 Cat# 5441; Sigma-Aldrich, Arklow, Ireland) diluted in 5% milk/0.05% TBST overnight at 4 °C. Post incubation period, the membrane was washed in washing buffer (0.1% TBST) for 3 × 10 min washes. After washing, membranes were then incubated in secondary antibody solution containing IR-Dye goat anti-mouse (k700) and goat anti-rabbit or donkey anti-goat (k800) (LI-COR Biosciences, Cambridge, UK) diluted 1:10,000 in 1% milk/0.1% TBST for 1 h. Five × 5 min washing steps were then performed with washing buffer (0.1% TBST) and one final 5 min wash in distilled water. Blots were scanned on a LI-COR Odyssey imager. IR band intensities for PPAR receptor protein expression (~52/55 kDa) for each sample were generated automatically using the background subtraction method of the LI-COR Image Studio Ver. 2.0 imaging software. Two distinct bands were observed for PPARγ (refer to Figure 15) due to the existence of two isoforms for this receptor. Because the antibodies for PPARα and PPARβ/δ were raised in mouse, similarly to β-actin, these two isoforms showed a band for the endogenous control taken in a second moment. The membranes were stripped of the binding of PPAR antibodies using a stripping buffer, and the protocol described above was repeated from the blocking in 5% non-fat dry milk in the 0.1% TBST step, and the membrane was then re-probed using β-actin antibodies. The blots were then re-scanned on a LI-COR Odyssey imager. IR band intensities for β-actin (~42 kDa) were generated automatically using the background subtraction method of the LI-COR Image Studio Ver. 2.0 imaging software.

#### 4.9.2. Verification of PPARβ/δ Expression in the BLA by RT-qPCR

Punched brain tissues from BLA of naïve male Sprague-Dawley rats were analysed by quantitative real-time PCR (RT-qPCR). RT-qPCR was carried out as described previously (68,87). RNA was extracted from BLA tissue (BLA: 2.04 mg ± 0.2 mg) using the Macherey-Nagel NucleoSpin ^®^ RNA Extraction Kit (Nucleospin RNA, Fisher Scientific, Dublin, Ireland), according to the instructions of the manufacturer. Tissue was homogenised in 353.5 µL of lysis buffer (RA1) containing β-mercaptoethanol (Sigma, Dublin, Ireland) for 3–5 s using an automated homogeniser (Polytron tissue disrupter, Ultra-Turrax, Staufen, Germany). Homogenates were kept on ice until transferred to a Nucleospin filter (violet ring) and centrifuged at 11,000× *g* for 1 min to reduce viscosity and clear the lysate. The lysates were then treated with 350 µL of 70% molecular grade ethanol (Sigma, Dublin, Ireland) and transferred to a Nucleospin RNA column (light blue ring) and centrifuged at 11,000× *g* for 30 s to bind the RNA to the membrane. The membrane column was then desalted by adding 350 µL of membrane desalting buffer (MDB) and centrifuging at 11,000× *g* for 1 min to dry the membrane. Samples were then treated with 10 μL rDNase and left for 15 min at room temperature to remove any DNA. Samples were then serially washed using washing buffers (200 µL RA2, 600 µL RA3, and 250 µL RA3) and RNA was eluted in 30 µL of RNAase-free water (Sigma, Dublin, Ireland). Nanodrop technology (ND-1000, Nanodrop, Labtech International, Ringmer, UK) was used to measure the concentration, purity, and integrity of the RNA. RNA concentration was determined by measuring the optical density (OD) at 260 nm. The integrity and purity were determined by measuring the ratios of OD260/OD280 and OD230/OD280, respectively, where a ratio of approximately 1.8–2.0 was deemed indicative of RNA of good quality and purity. All RNA samples were within the acceptable range for both integrity and purity. Samples were equalised to the same concentration of RNA (35 ng/μL) using RNase free water (Sigma, Dublin, Ireland). Equalised samples were then stored at −80 °C until reverse transcribed. Equal amounts of total RNA (10 ng/μL) were reverse transcribed into cDNA as follows: two master mixes were made up, as shown below in Supplementary Tables S2 and S3; all reagents were obtained from (Biosciences, Dublin, Ireland). Ten μL of normalised RNA from each sample was added to a newly labelled PCR tube where 2 μL of master mix 1 was added to each tube. The mixture was then heated to 65 °C for 5 min in a thermocycler (MJ Research, Reno, NV, USA) and quickly chilled on ice. The contents of the tube were collected by brief centrifugation. Seven μL of master mix 2 was then added to each tube and incubated at 37 °C for 2 min on the thermocycler. One μL of superscript III reverse transcriptase was added to each sample and mixed gently. Samples were left to incubate at room temperature for 10-min and then loaded on the thermocycler to incubate further at 50 °C for 50 min. The reaction was inactivated by heating the samples at 70 °C for another 15 min. Finally, cDNA samples were diluted (1:4) using RNAase-free water and stored at −20 °C. cDNA strands were then analysed by RT-qPCR using the Applied Biosystems StepOne Plus Real Time PCR System (Bio-Sciences, Dublin, Ireland). TaqMan gene expression assays (Bio-Sciences, Dublin, Ireland) containing forward and reverse primers and a FAM-labelled TaqMan probe were used (Bio-Sciences, Dublin, Ireland). Assay IDs for the genes in rats examined were as follows: PPARβ/δ (Rn00565707) and VIC-labelled β-actin (Rn00667869_m1) was used as the house keeping gene and endogenous control. A reaction mixture was prepared and stored on ice. This consisted of 0.5 μL target (PPAR) primers (Bio-Sciences, Dublin, Ireland), 0.5 μL of the reference gene β-actin, 5 μL TaqMan Universal PCR master mix, 1.5 μL of RNA free water, and 2.5 μL of sample cDNA to give a total volume of 10 μL per sample. Samples were pipetted in duplicate (10 μL per well total volume) into an optical 96 well plate. Negative controls were included in all assays, containing the master mix but cDNA was replaced with RNase free water. Plates were then covered with adhesive covers and spun at 1000 g for 1 min to ensure complete mixing. The plate was then placed in a StepOnePlus™ real time PCR machine (Bio-Sciences, Dublin, Ireland). StepOnePlus™ cycling conditions were 50 °C for 2 min, 95 °C for 10 min, and 40 cycles of (95 °C for 15 s/60 °C for 1 min). Amplification plots were examined using Applied Biosystems 7500 System SDS Software 1.3.1.

### 4.10. Statistical Analysis

The SPSS 21.0 statistical package was used to analyse the data. Normality was assessed using thee Shapiro–Wilk test and homogeneity of variance was checked using Levene’s test. Behavioural data were analysed using two-factor analysis of variance (two-way ANOVA), with factors being fear-conditioning and treatment, or analysis of variance with repeated measures (repeated measures ANOVA) when appropriate (e.g., when the data were analysed and presented in time bins). Neurochemical data were analysed using three-factor analysis of variance (three-way ANOVA), with factors being fear conditioning, treatment, and side (ipsilateral or contralateral, with respect to the formalin injection). Post hoc pairwise comparisons were made with Student Newman–Keuls test when appropriate. If data were found to be non-parametric, three transformation protocols were applied, in this order: square root of the data values, log of the data values, and ranking of the data values. Additionally, we checked whether the highest standard deviation was less than or equal to two times the smallest standard deviation for the particular dataset being analysed [89]. If data were still deemed non-parametric after these transformations and tests, they were analysed using Kruskal–Wallis analysis of variance and post hoc analysis performed using Dunn’s test when appropriate. When repeated measures data were non-parametric, they were analysed using Friedman’s and Kruskal–Wallis tests, followed by Dunn’s post hoc if applicable. Data were considered significant when *p* < 0.05. Data are expressed as group means ± standard error of the mean (S.E.M.) when parametric and as median with interquartile range and min/max when non-parametric.

## Data Availability

The data presented in this study are available on request from the corresponding author.

## Supplementary Materials

The following supporting information can be downloaded at: https://www.mdpi.com/article/10.3390/molecules27062021/s1, Figure S1: Histological verification of injector site location for Experiment 1; Figure S2: Effects of intra-BLA administration of selective PPARα, PPARβ/δ, and PPARγ antagonists on freezing duration presented as 3-min time bins (B) in non-fear conditioned (NFC) rats; Figure S3: Histological verification of injector site location for Experiment 1; Figure S4: Effects of intra-BLA administration of selective PPARα, PPARβ/δ, and PPARγ antagonists on freezing duration presented as 3-min time bins in fear conditioned (FC) and non-fear conditioned (NFC) rats; Table S1: Summary of experimental groups. NFC, non-fear conditioned; FC, fear conditioned; Table S2: Supplementary Table S1: Master mixture 1 for cDNA synthesis; Table S3: Supplementary Table S3: Master mixture 2 for cDNA synthesis; Annex S1: Description of statistical results for Section 2.4.1; Annex S2: Description of statistical results for Section 2.4.2.

## Sample Availability

Laboratory samples may be discarded following publication of this work.
