# Supplementary material for: Effects of Intra-BLA Administration of PPAR Antagonists on Formalin-Evoked Nociceptive Behaviour, Fear-Conditioned Analgesia, and Conditioned Fear in the Presence or Absence of Nociceptive Tone in Rats
Source: Molecules. 2022 Mar 21;27(6):2021. doi: 10.3390/molecules27062021 (PMC8949000; doi:10.3390/molecules27062021)
Supplement: Supplementary file 1 [file molecules-27-02021-s001.zip › molecules-1602007-supplementary.pdf]

molecules

Supplementary Materials

Effects of Intra-BLA Administration of PPAR Antagonists on Formalin-Evoked Nociceptive Behaviour, Fear-Conditioned Analgesia, and Conditioned Fear in the Presence or Absence of Nociceptive Tone in Rats

Jessica C. Gaspar [1,2,3], Bright N. Okine [1,2,3], David Dinneen [1,3,4], Michelle Roche [2,3,4] and David P. Finn [1,2,3,5,*]

1 Pharmacology and Therapeutics, National University of Ireland Galway, Galway, Ireland; H91 W5P7 jeccgaspar@gmail.com (J.C.G.); bnokine@gmail.com (B.N.O.); ddinneen01@qub.ac.uk (D.D.)
2 Galway Neuroscience Centre, National University of Ireland Galway, Galway, Ireland; michelle.roche@nuigalway.ie
3 Centre for Pain Research, National University of Ireland Galway, Galway, Ireland
4 Physiology, National University of Ireland Galway, Galway, Ireland
5 Pharmacology and Therapeutics, Human Biology Building, National University of Ireland Galway, University Road, Galway, Ireland
* Correspondence: david.finn@nuigalway.ie; Tel.: +353-(0)91-495280

Supplementary Table S1. Summary of experimental groups. NFC, non-fear conditioned; FC, fear conditioned.

Experiments	Intraplantar injection	Treatment	Conditioning	
			NFC (n per group)	FC (n per group)
**Experiment 1**	**Formalin**	Vehicle	11	11
	Formalin	GW6471 (PPARα antagonist)	11	11
	Formalin	GSK0660 (PPARβ/δ antagonist)	11	11
	Formalin	GW9662 (PPARγ antagonist)	11	11
Experiment 2	Saline	Vehicle	11	11
	Saline	GW6471 (PPARα antagonist)	11	11
	Saline	GSK0660 (PPARβ/δ antagonist)	11	11
	Saline	GW9662 (PPARγ antagonist)	11	11

Supplementary Table S2. Master mixture 1 for cDNA synthesis.

Reagents	Per Sample
Random Primers (250ng)	1µl
10mm dNTP mix	1µl
**Total**	**2µl**

Supplementary Table S3. Master mixture 2 for cDNA synthesis.

Reagents	Per Sample
5X First Strand Buffer	4µl
0.1M DTT	2µl
RNase Out	1µl
**Total**	**7µl**

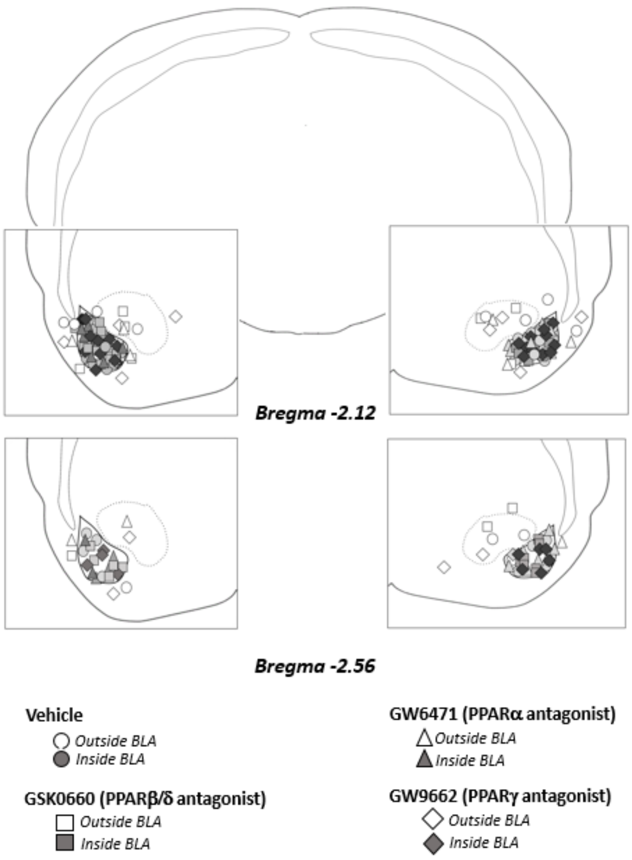

Supplementary Figure S1. Histological verification of injector site location for experiment 1.

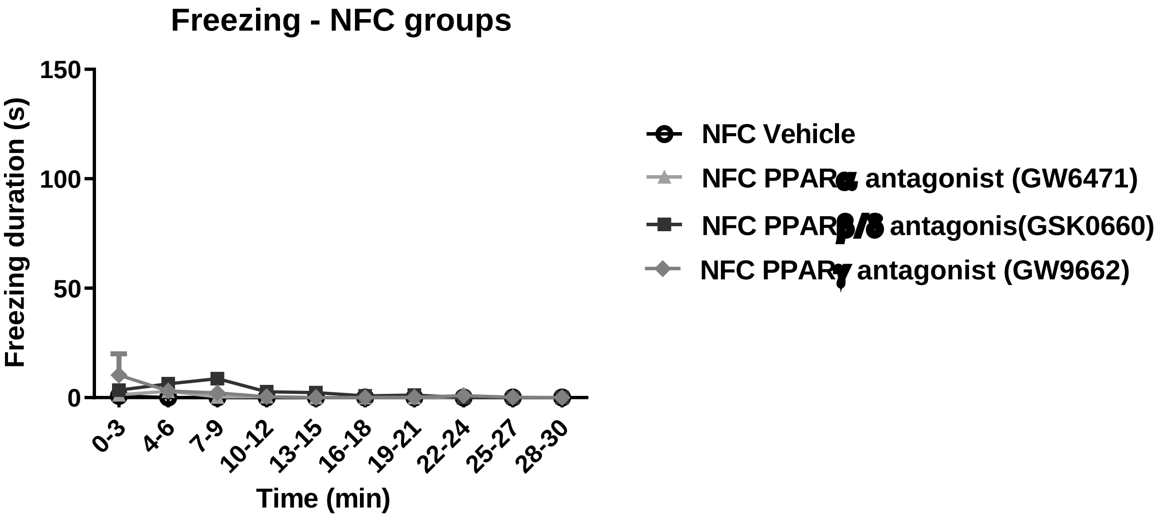

Supplementary Figure S2. Effects of intra-BLA administration of selective PPARα, PPARβ/δ and PPARγ antagonists on freezing duration presented as 3-min time bins (B) in non-fear conditioned (NFC) rats.

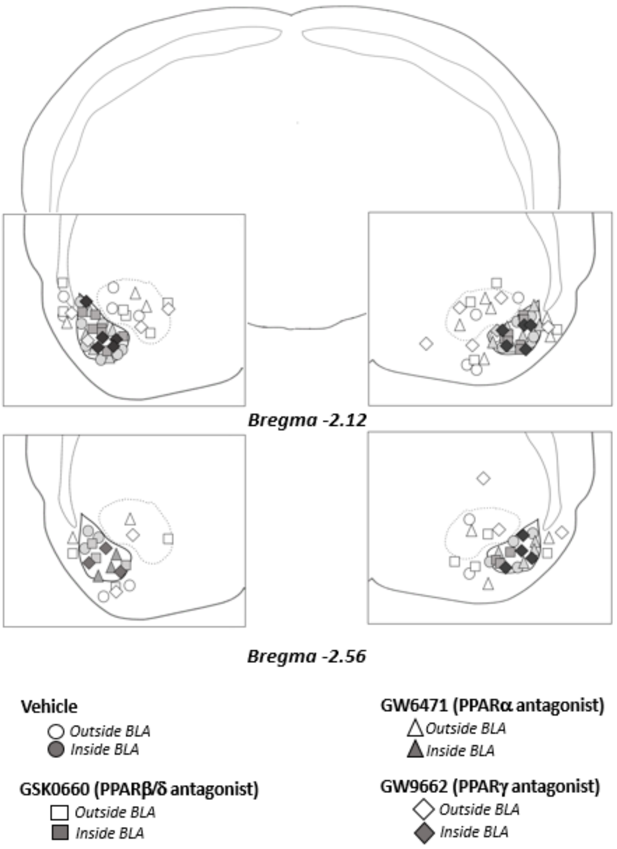

Supplementary Figure S3. Histological verification of injector site location for experiment 2.

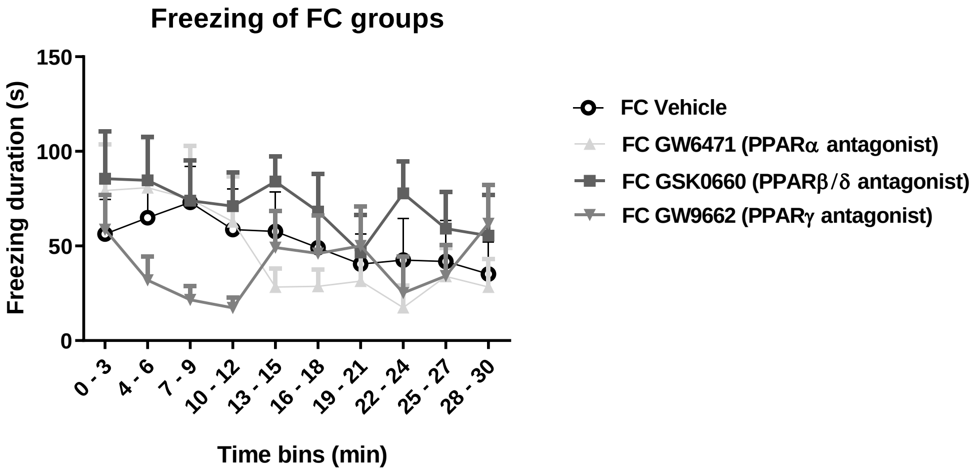

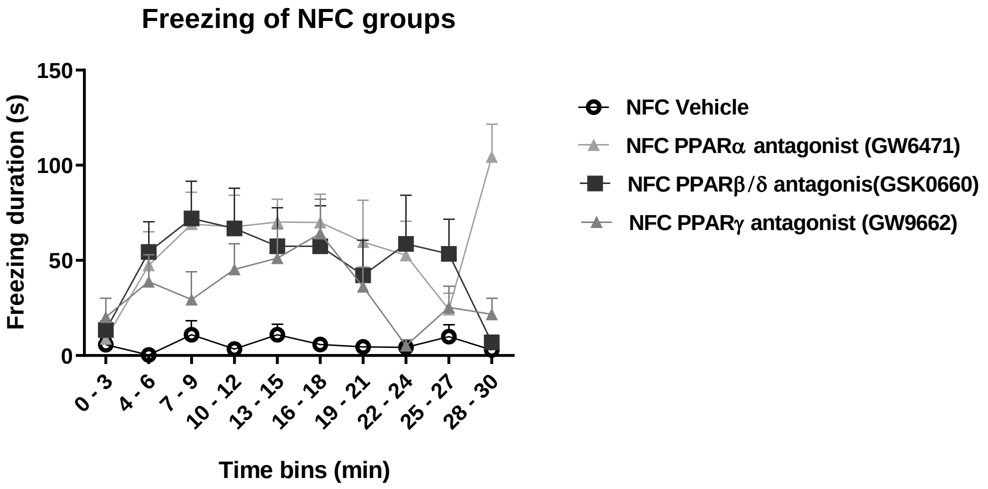

Supplementary Figure S4. Effects of intra-BLA administration of selective PPARα, PPARβ/δ and PPARγ antagonists on freezing duration presented as 3-min time bins in fear conditioned (FC) or non-fear conditioned (NFC) rats.

Annex S1 – Description of statistical results for section 2.4.1

Three-way ANOVA revealed a significant main effect of side [F (1, 52) = 10.730, ^{a}p=0.002] on GABA levels in the BLA (Figure 17A). *Post hoc* pairwise analysis with Student Newman-Keuls did not show any significant statistical differences. There were no significant effects of fear conditioning, treatment [F (1, 52) = 2.446, p>0.05], treatment x conditioning [F (1, 52) = 2.030, p>0.05], treatment x side [F (1, 52) = 0.022, p>0.05], conditioning x side [F (1, 52) = 0.556, p>0.05], treatment x conditioning x side [F (1, 52) = 3.365, p>0.05] on GABA levels. When the right and left sides were analysed separately, two-way ANOVA did not show any significant effect of treatment, conditioning or their interaction on GABA levels in either left or right BLA.

Three-way ANOVA revealed an effect of side [F (1, 52) = 5.630, ^{a}p=0.021] on glutamate levels in the BLA (Figure 17B). *Post hoc* pairwise analysis with Student-Newman-Keuls did not show any significant statistical differences. There were no significant effects of fear conditioning [F (1, 52) = 0.103, p>0.05], treatment [F (1, 52) = 0.865, p>0.05], treatment x conditioning [F (1, 52) = 1.429, p>0.05], treatment x side [F (1, 52) = 0.637, p>0.05], conditioning x side [F (1, 52) = 0.007, p>0.05], treatment x conditioning x side [F (1, 52) = 1.133,

p>0.05] on glutamate levels. When the contra and left sides were analysed separately, two-way ANOVA did not show any significant effect of treatment, conditioning or their interaction on glutamate in either left or right BLA.

Three-way ANOVA revealed an effect of side [F (1, 51) = 12.192, [a]p=0.001] and fear conditioning [F (1, 51) = 5.238, p=0.026] on serotonin levels in the BLA (Figure 17C). *Post hoc* pairwise analysis with Student Newman-Keuls indicated that saline-treated FC rats have increased levels of serotonin compared to their NFC counterparts (NFC Saline-treated vs FC Saline-treated, [#]p<0.05) on the right side. There were no significant effects of treatment [F (1, 51) = 0.029, p>0.05], treatment x conditioning [F (1, 51) = 1.564, p>0.05], treatment x side [F (1, 51) = 1.644, p>0.05], conditioning x side [F (1, 51) = 2.044, p>0.05], treatment x conditioning x side [F (1, 51) = 3.796, p>0.05] on serotonin levels. When right and left sides were analysed separately, two-way ANOVA revealed significant effect of fear conditioning [F (1, 24) = 4.464, [£]p<0.05] on serotonin levels in the right BLA. However, p*ost hoc* pairwise analysis with Student Newman-Keuls did not show significant statistical differences. Two-way ANOVA showed that there were no significant effects of treatment, conditioning and their interaction on serotonin levels in the left BLA.

Three-way ANOVA revealed an effect of side [F (1, 47) = 53.882, [a]p<0.001] and treatment [F (1, 47) = 14.541, p<0.001] on dopamine levels in the BLA (Figure 17D). *Post hoc* pairwise analysis with Student Newman-Keuls confirmed the side differences (*p<0.05, compared to their left counterparts) and indicated that NFC rats which received an intraplantar injection of formalin have increased levels of dopamine on the right BLA (NFC Saline-treated vs NFC-Formalin-treated, [#]p<0.05). There were no significant effects of fear conditioning [F (1, 47) = 0.002, p>0.05], treatment x conditioning [F (1, 47) = 0.055, p>0.05], treatment x side [F (1, 47) = 2.115, p>0.05], conditioning x side [F (1, 47) = 0.477, p>0.05], treatment x conditioning x side [F (1, 47) = 1.358, p>0.05] on dopamine levels. When the right and left sides were analysed separately, two-way ANOVA did not show any significant effect of treatment, conditioning or their interaction on dopamine levels in either left or right BLA.

Annex S2 – Description of statistical results for section 2.4.2

Three-way ANOVA revealed an effect of side [F (1, 49) = 4.191, [a]p=0.046] on PEA levels in the BLA (Fig. 18A). *Post hoc* pairwise analysis with Student Newman-Keuls did not show significant statistical differences. There were no significant effects of fear conditioning [F (1, 49) = 3.237, p>0.05], treatment [F (1, 49) = 3.912, p>0.05], treatment x conditioning [F (1, 49) = 0.035, p>0.05], treatment x side [F (1, 49) = 3.758, p>0.05], conditioning x side [F (1, 49) = 0.856, p>0.05], treatment x conditioning x side [F (1, 49) = 1.275, p>0.05] on PEA levels. When right and left sides were analysed separately, two-way ANOVA revealed significant effect of treatment [F (1, 23) = 8.216, p=0.009] on PEA levels in the right BLA. *Post hoc* pairwise analysis with Student-Newman-Keuls indicated that FC rats that received formalin injection had lower levels of PEA in the right side compared to their saline-treated counterparts (FC Formalin-treated vs FC Saline-treated, [$]p<0.05). Two-way ANOVA showed that there were no significant effects of treatment, conditioning or their interaction on PEA levels in the left BLA.

Kruskal-Wallis comparisons revealed a significant difference among groups (χ^2 (7) = 35.131, p<0.05) in AEA levels (Figure 18B). *Post hoc* analysis with Dunn's test showed lower levels of AEA of NFC Saline group in the right side compared to the left (*p<0.05). When each side was analysed separately, Kruskal Wallis did not reveal any significant differences among group in the right [χ^2 (3) =6.485, p>0.05] or in the left [χ^2 (3) = 2.456, p>0.05] side.

Three-way ANOVA revealed an effect of side [F (1, 48) = 9.699, [a]p=0.003] on OEA levels in the BLA (Fig. 18C). *Post hoc* pairwise analysis with Student Newman-Keuls did not show significant statistical differences. There were no significant effects of fear conditioning [F (1, 48) = 3.013, p>0.05], treatment [F (1, 48) = 0.346, p>0.05], treatment x conditioning [F (1, 48) = 0.087, p>0.05], treatment x side [F (1, 48) = 2.259, p>0.05], conditioning

x side [$F(1, 48) = 0.308$, $p>0.05$], treatment x conditioning x side [$F(1, 48) = 1.667$, $p>0.05$] on OEA levels. When the right and left sides were analysed separately, two-way ANOVA showed that there were no significant effects of treatment, conditioning or their interaction on OEA levels in either the left or right BLA.
